# Metformin causes a futile intestinal–hepatic cycle which increases energy expenditure and slows down development of a type 2 diabetes-like state

**DOI:** 10.1016/j.molmet.2017.05.002

**Published:** 2017-05-05

**Authors:** Philipp Schommers, Anna Thurau, Insa Bultmann-Mellin, Maria Guschlbauer, Andreas R. Klatt, Jan Rozman, Martin Klingenspor, Martin Hrabe de Angelis, Jens Alber, Dirk Gründemann, Anja Sterner-Kock, Rudolf J. Wiesner

**Affiliations:** 1 Institute of Vegetative Physiology, Medical Faculty, University of Köln, 50931 Köln, Germany; 2 Department I of Internal Medicine, University Hospital Cologne, 50931 Köln, Germany; 3 Center for Experimental Medicine, Medical Faculty, University Hospital Cologne, 50931 Köln, Germany; 4 Institute for Clinical Chemistry, Medical Faculty, University Hospital Cologne, 50931 Köln, Germany; 5 German Mouse Clinic, Helmholtz Zentrum München, German Research Center for Environmental Health, 85764 Neuherberg, Germany; 6 German Center for Diabetes Research (DZD), 85764 Neuherberg, Germany; 7 Chair of Molecular Nutritional Medicine, Technische Universität München, Else Kröner-Fresenius Center for Nutritional Medicine, 85350 Freising, Germany; 8 ZIEL – Institute for Food and Health, Technische Universität München, 85350 Freising, Germany; 9 Max-Planck Institute for Metabolism Research, 50931 Köln, Germany; 10 Department of Pharmacology, Medical Faculty, University of Köln, 50931 Köln, Germany; 11 Center for Molecular Medicine Cologne (CMMC), University of Köln, 50931 Köln, Germany; 12 Cologne Excellence Cluster on Cellular Stress Responses in Aging-associated Diseases (CECAD), University of Köln, 50931 Köln, Germany

**Keywords:** Futile cycle, Splanchnic bed, Metformin, Mitochondria

## Abstract

**Objective:**

Metformin, the first line drug for treatment of type 2 diabetes, suppresses hepatic gluconeogenesis and reduces body weight in patients, the latter by an unknown mechanism.

**Methods:**

Mice on a high fat diet were continuously fed metformin in a therapeutically relevant dose, mimicking a retarded formulation.

**Results:**

Feeding metformin in pharmacologically relevant doses to mice on a high fat diet normalized HbA1c levels and ameliorated glucose tolerance, as expected, but also considerably slowed down weight gain. This was due to increased energy expenditure, since food intake was unchanged and locomotor activity was even decreased. Metformin caused lactate accumulation in the intestinal wall and in portal venous blood but not in peripheral blood or the liver. Increased conversion of glucose-1-^13^C to glucose-1,6-^13^C under metformin strongly supports a futile cycle of lactic acid production in the intestinal wall, and usage of the produced lactate for gluconeogenesis in liver.

**Conclusions:**

The reported glucose–lactate–glucose cycle is a highly energy consuming process, explaining the beneficial effects of metformin given continuously on the development of a type 2 diabetic-like state in our mice.

## Highlights

• Orally administered metformin slowed down weight gain on a high fat diet.
• Metformin treatment led to increased energy expenditure, but decreased locomotion.
• Metformin treatment caused a futile, energy consuming glucose–lactate–glucose cycle.

## 1 Introduction

Type 2 Diabetes Mellitus (T2DM) is one of the most common disorders in industrialized countries, with rapidly increasing patient numbers in the last decades; thus, its successful treatment in the setting of a metabolic syndrome gets more and more important. According to the 2015 guidelines of the ADA (American Diabetes Association) and the EASD (European Diabetes Association), lifestyle modification, i.e. weight control and physical activity, in combination with metformin (1,1-dimethylbiguanide) is the current first-line therapeutic concept for T2DM patients [1], [2]. Basically, metformin lowers elevated blood glucose levels, and, with the successful treatment of hyperglycemia, it results in a significantly reduced diabetes-related morbidity [3]. Beyond its glucose lowering effect, metformin-treatment results in significant weight loss (summarized in recent large meta-analyses [4], [5]). Surprisingly, the mechanisms responsible for lowering body weight are unknown, even though weight loss alone improves glucose homeostasis in T2DM.

Although being introduced and available for clinical use since the 1950ies, metformin's therapeutic mechanisms are still not understood. One of the earliest possible modes of action, then identified for alkylguanidines that are closely related to the biguanidine metformin, was inhibition of oxygen consumption in liver mitochondria [6]. Later, it was shown in isolated hepatocytes that metformin in high concentrations specifically inhibits complex I of the respiratory chain and reduces gluconeogenesis, probably by inhibiting pyruvate carboxylase inside mitochondria, a rate limiting enzyme of this process which is sensitive to the cellular energy as well as the redox state [7], [8]. Stimulated by the finding that the ATP/ADP ratio was lowered by metformin in liver, the AMP-activated protein kinase (AMPK) was subsequently shown to be activated by metformin in isolated hepatocytes [9]. However, later it was shown that in mice lacking both isoforms of the AMPKα subunit as well as the upstream kinase LKB1, the hypoglycemic effect of the drug was still maintained [10], [11]. Since then, new alternative mechanisms explaining how metformin may inhibit hepatic gluconeogenesis have been proposed, e.g. suppression of glucagon signaling by interfering with cAMP production [12], altering the hepatic redox state by direct inhibition of mitochondrial glycerophosphate dehydrogenase [13] as well as activation of a neurohumoral gut–brain–liver axis [14].

Most studies still concentrate on the liver as the main target of metformin, arguing that this organ plays the key-role in gluconeogenesis and that intracellular drug concentrations will reach high levels after orally administered metformin is absorbed by the intestine [15]. It was previously shown that an important mode-of-action of the drug is to improve lipid homeostasis by stimulating the AMPK mediated phosphorylation of acetyl-CoA carboxylases, which consequently improves insulin sensitivity [16]. Although elegant, it is important to note that the drug was applied by intraperitoneal injection, thus bypassing the physiological route of orally taken metformin.

However, after oral administration, the highest concentration of metformin is not found in the liver but in the intestinal epithelium [17], [18], [19]. Early data using obese fa/fa rats already showed that metformin administration significantly increased glucose consumption in the intestine [20] due to mitochondrial inhibition, which was confirmed later demonstrating increased lactate production in isolated human jejunal preparations [19].

Here, we continuously fed metformin in a therapeutically relevant dose, mimicking a retarded formulation, to mice on a high fat diet in order to investigate how the drug slows down the development of T2DM, but most importantly, how it slows down weight gain, the other well described mode of action in patients.

## 2 Materials and methods

### 2.1 Animals and experimental protocols

Animals (male C57BL6/J mice) were housed in a 12 h light-dark circle (06:00 on, 18:00 off, including a period of dawn) at constant temperature of 22 °C and a humidity of 60 rH.

Controls received standard chow (Altromin Spezialfutter, Lage, Germany, #TPF-1314: 5% fat, 4.8% disaccharide, 23% protein). HFD treated mice were fed a high fat, high sucrose diet (HFD; Altromin Spezialfutter, Lage, Germany, #105712: 35% fat, 19% disaccharide, 19% protein). HFD + Met and HFD + lateMet received the same HFD supplemented with 0,5% of metformin (1,1-Dimethylbiguanide Hydrochloride, 97%, Sigma Aldrich, Darmstadt, Germany, #D150959). Metformin was added to the diet during the manufacturing process to assure equal distribution over the HFD. Mice had access to chow and water ad libitum unless otherwise specified.

A detailed description about the different cohorts of mice used in this study can be found in the supplementary experimental procedures. All animal procedures were performed in accordance with the German Laws for Animal Protection and were approved by the local animal care committee (Landesamt für Natur-, Umwelt und Verbraucherschutz, LANUV, Recklinghausen, Germany; Az 37.09.298).

### 2.2 Metabolic characterization and glucose tolerance test

Mice were weighed every two weeks, starting at 6 weeks of age. Intraperitoneal glucose tolerance tests (GTT) were performed every 2 weeks, starting at 12 weeks of age, after 16 h of fasting by an intraperitoneal injection of 2 g/kg glucose (Supplementary Figure 1). Glucose was measured in tail venous blood at 15, 30, 60 and 120 min after injection (Glucomen LX, Berlin-Chemie, Berlin, Germany).

### 2.3 Indirect calorimetry and physical activity measurement

At the age of 6 weeks, this cohort of animals was divided into two groups and fed HFD + Met or HFD (n = 16 each) for 12 weeks. At the age of 18 weeks, mice were kept for 48 h (starting at 10 am) in an open circuit measurement system (PhenoMaster, TSE Systems GmbH, Bad Homburg, Germany) after having been acquainted to the new environment. Light phase was from 7:30 PM until 6:30 AM. The following parameters were obtained: CO_2_ production, O_2_ consumption, home cage activity, food intake, water intake and feeding events, and values for energy expenditure (EE) and respiratory exchange ratio (RER; CO_2_ production/O_2_ consumption) were derived from these measurements.

### 2.4 Inhibition of mitochondrial complex I and integrity of the intestinal mucosa in the presence of metformin

Mice were sacrificed by cervical dislocation; the small intestine (duodenum) was removed and immediately cooled on a metal plate kept on ice to slow down self-digestion as much as possible. Samples from the duodenum were immersed in Tissue Tek (O.C.T.™ Compound – Sakura Finetek, Staufen, Germany), immediately frozen in liquid nitrogen, and sectioned using a Leica CM1950 cryostat (Leica Microsystems, Wetzlar, Germany) at −20 °C. In order to quantitate NADH dehydrogenase (Complex I) activity, the following method was used (Diaphorase activity, modified from [21]: directly after preparation, 2–3 μm sections were incubated in 0.1 M Tris, pH 7.4, 4 mM NADH, 0.1 M Tris–HCl, pH 7.4, 0.2 mM nitroblue tetrazolium chloride for 10 min at room temperature. Sections were then briefly rinsed with distilled water, dried, and mounted in aqueous medium. Sections incubated without NADH were used as controls. Before, it was established that after 10 min, staining had not reached maximal intensity, thus allowing determination of Complex I activity *in situ* in different samples processed in a highly parallel way. Images were taken at 20× or 40× magnification using an Olympus BX-40 microscope (Olympus, Hamburg, Germany) and staining intensity was analyzed using a thresholding tool-based method (Using ImageJ [22], and the image processing package Fiji for Image J [23], [24].). The thresholding tool settings were established in samples from HFD mice and used for quantification of all samples. The ratio of staining-positive to total area of 2–4 samples of the duodenum from HFD and HFD + Met mice (n = 2) were determined.

Staining procedures for hematoxylin-eosin (HE) and PCNA (Proliferating cell nuclear antigen) are described in the supplementary experimental procedures.

### 2.5 Quantitation of glucose, lactate, and metformin by LC-MS/MS

Mice were fed with HFD or HFD + Met lacking disaccharides, that was supplemented with 1 g of glucose-1-^13^C (# 297046; Sigma Aldrich, Darmstadt, Germany) per 10 g of food. After 3 h, cheek punch blood samples of variable size were collected directly in 100 μl acetonitrile and diluted 1:10 with acetonitrile; serum samples generated from ventricular cavity blood were diluted 1:10 or 1:100 with acetonitrile. Of these, 10 or 20 μl were analyzed on a triple quadrupole mass spectrometer (4000 QTRAP, AB Sciex, Darmstadt, Germany). The following LC conditions were used (Shimadzu SLC-20AD Prominence HPLC, Kyoto, Japan): for glucose and lactate, SeQuant ZIC-pHILIC column (5 μm, 2.1 × 100 mm; Dichrom, Marl, Germany), A 0.1% ammonia, B acetonitrile, gradient flow 0.2 ml/min, 70% B at 0 min, 10% B at 4 min, 10% B at 7 min, 70% B at 10 min, stop at 11 min; for methionine, SeQuant ZIC-HILIC column (5 μm, 2.1 × 100 mm), A 0.1% formic acid, B 0.1% formic acid in acetonitrile, isocratic flow 0.3 ml/min, 40% B, stop at 4 min; for metformin, Atlantis HILIC column (5 μm, 3 × 50 mm; Waters, Eschborn, Germany), A 10 mM ammonium formate pH 3.8, B methanol, gradient flow 0.4 ml/min, 80% B at 0 min, 20% B at 2 min, 20% B at 4 min, 80% B at 6 min, stop at 8 min. Atmospheric pressure ionization with positive or negative electrospray was used. The following fragments were chosen for selected reaction monitoring (*m*/*z* parent, *m*/*z* fragment, collision energy (V; minus indicates negative ion detection)): glucose, 179, 89, −10; glucose-1-^13^C, 180, 90, −12; glucose-1,6-^13^C, 181, 90, −30; lactate, 89, 43, −20; lactate-3-^13^C, 90, 44, −13; metformin, 130, 60, 19; methionine, 150, 133, 15. For each analyte, the peak area was obtained from integrating intensity above background vs. time in the proper elution time interval. Methionine was chosen to normalize the cheek punch data because here the peak area followed dilution steps best. However, similar results were obtained with leucine, phenylalanine, and valine. Serum data were not normalized.

### 2.6 Further experimental procedures

Procedures describing immunoblotting, qRT-PCR and determination of energy content of feces are described in detail in the supplementary experimental procedures. In short, pACC/ACC ratios and quantification of Ucp1 expression in BAT and WAT were determined by immunoblotting of proteins from liver samples after 12 weeks of treatment. Expression of monocarboxylate transporters and lactate dehydrogenase isoforms were analyzed by qRT-PCR. Analysis of the energy content of feces was performed by Fourier-transformed infrared (FT/IR) reflectometry and following bomb calorimetry.

### 2.7 Statistical analysis

All data are presented as means ± S.D. Areas under the curve (AUC) were calculated using the trapezoidal method [25]. Differences between groups were analyzed by Student's *t*-test or 1-way ANOVA, followed by Bonferroni correction, as appropriate (see Figure legends). Statistical significance of post-hoc analyses was accepted at p < 0.05. Calculations were performed using SPSS21 (IBM Deutschland, Ehningen, Germany).

## 3 Results

### 3.1 Continuous metformin application slows down body weight gain

Based on an extended pre-experiment, four groups were established (Supplementary Figure 1). In one group, metformin was given to mice that had already developed glucose intolerance (HFD + lateMet); the other group was treated together with the onset of HFD feeding (HFD + Met). Considering the food intake of our mice (about 0.08 g g^−1^ body weight per day of the HFD + 0,5% metformin diet), daily metformin intake was about 500 mg kg^−1^ per day, which equates to a human equivalent dose (HED) [26] of about 40 mg metformin kg^−1^ per day. Extrapolated to the average patient's weight, this dose is in the same range as the maximum dose of metformin given to humans (3 × 1000 mg per day) [27].

**Figure 1:**
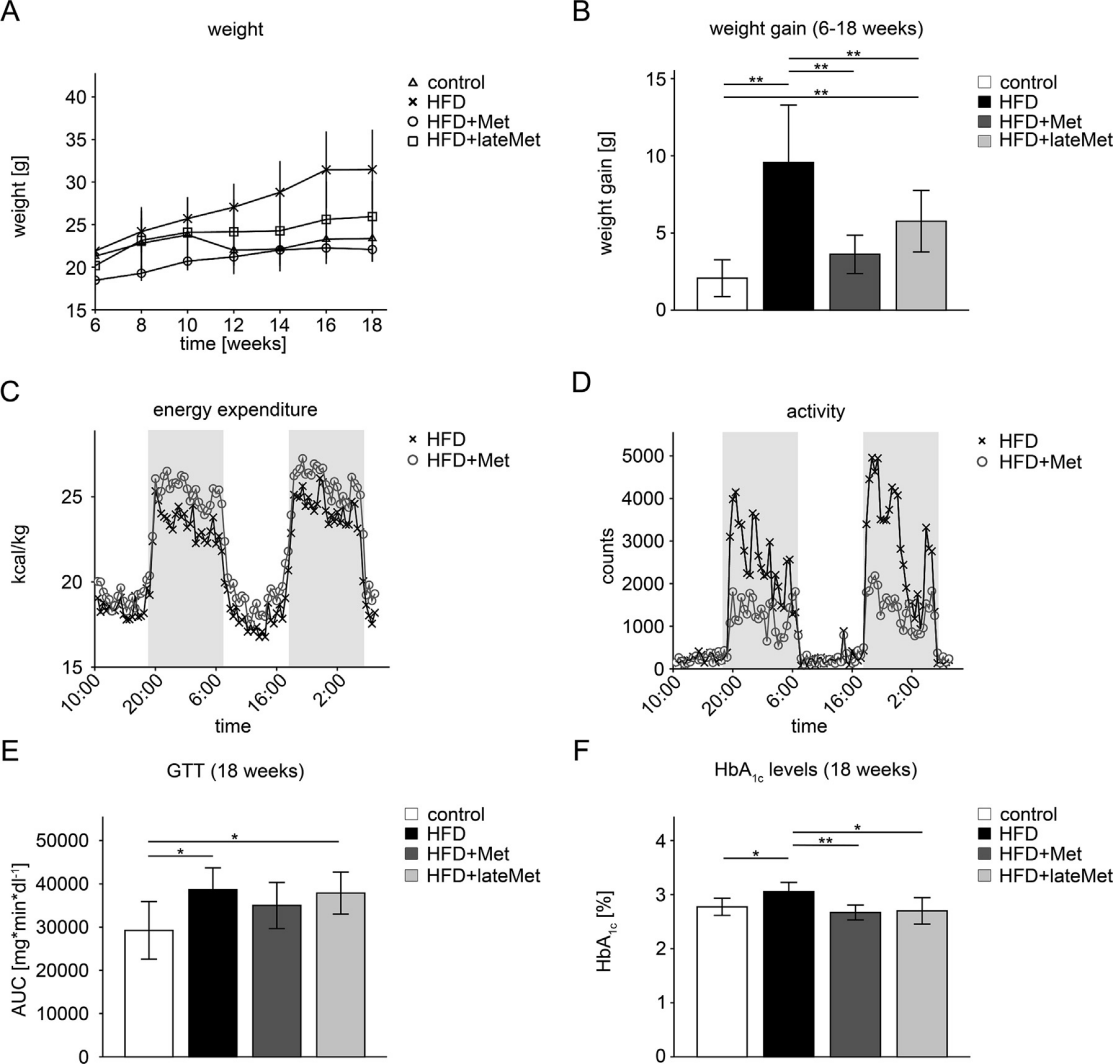
**Metformin slowed down the development of obesity and type 2 diabetes in mice upon a high-fat diet and increased energy expenditure**. (A + B) Compared to mice fed with high fat diet (HFD; n = 8), metformin (Met) treatment significantly slowed down weight gain in mice fed with high fat diet and treated with metformin starting at the same time (HFD + Met; n = 11; p < 0.01) and HFD mice treated with metformin after already developing glucose intolerance (HFD + lateMet; n = 8; p < 0.01) mice. Control mice (n = 11) received standard chow and their weight gain was considered as normal (p < 0.01 vs. HFD and HFD + lateMet, respectively). (C + D) Indirect calorimetry and physical activity measurements after 12 weeks of treatment showed that (C) energy expenditure was persistently higher in HFD + Met (n = 16) mice, whereas (D) their locomotor activity was significantly lower during dark phases compared to HFD mice (n = 16). For statistical analysis of energy expenditure and activity, see Supplementary Figure S2. (E) Results of glucose tolerance tests (GTTs) at 18 weeks treatment are shown as areas under the curve. There were no differences in glucose tolerance between control (n = 10) and HFD + Met (n = 11) mice (p = n.s.). Glucose tolerance of HFD (n = 7) and HFD + lateMet (n = 8) was significantly higher compared to control mice (p < 0.05). (F) Compared to HFD (n = 7) mice, glycemic control, judged by glycated hemoglobin A1c (HbA1c) levels, was significantly improved in control (p < 0.05; n = 9), HFD + Met (p < 0.01; n = 9) and HFD + lateMet (p < 0.05; n = 8) mice. There were no differences between HFD + Met, HFD + lateMet and control mice (p = n.s.). Data are expressed as mean ± SD; for clarity, SDs are not shown in Figure 1 (C) and (D). n indicates the number of analyzed mice or of individual mice from which blood was collected and analyzed. Differences between groups were analyzed by 1-way ANOVA, followed by Bonferroni's post-hoc test; *p < 0.05 and **p < 0.01.

Weight development of the four groups is shown in Figure 1A. For better illustration and quantitation, we compared the increase of weight between 6 and 18 weeks (Figure 1B). While the HFD group gained 9.6 g, metformin considerably reduced weight gain, with only 5.8 g weight gain in the HFD + lateMet group and even only 3.6 g in the HFD + Met group (p < 0.01 vs. HFD, respectively).

### 3.2 Continuous metformin application increases energy expenditure

In order to find out how metformin treatment results in reduced weight gain, which is probably equivalent to weight loss in patients starting treatment and equivalent to improved maintenance of weight during treatment [28], [29], whole body metabolism was analyzed at the age of 18 weeks, after 12 weeks of HFD vs. HFD + Met. Reduced weight gain cannot be explained by decreased food intake, since this was similar in both groups, even though mice consuming metformin weighed less (HFD: 0.08 ± 0.03 g/day × g body weight vs. HFD + Met: 0.09 ± 0.02 g/day × g body weight; p = 0.29; Supplementary Figure 2A). However, energy expenditure of the HFD + Met mice was significantly higher, no matter if calculated from data for the entire 48 h period (Figure 1C; HFD: 21.1 ± 4.0 kcal/h × kg body weight vs. HFD + Met: 22.5 ± 4.9 kcal/h × kg body weight; p < 0.001) or by separating it into light and dark phases (Supplementary Figure 2B). Food, and thus also metformin, was consumed mostly during the dark phase (HFD: 18.13 ± 2.97 vs. HFD + Met: 19.13 ± 2.42 feeding events; p = n.s.), but also quite regularly during the light phase (HFD: 9.5 ± 3.3 vs. HFD + Met: 8.75 ± 2.3 feeding events; p = n.s.). Higher energy expenditure cannot be explained by increased locomotor activity, since HFD + Met mice were even less active (Figure 1D; HFD: 1524 ± 2182 counts/30 min vs. HFD + Met: 812 ± 688 counts; p < 0.001), mainly caused by a higher activity of the HFD group during the dark phase (Supplementary Figure 2C).

Also the respiratory exchange ratio (RER: CO_2_ production vs. O_2_ consumption) was slightly higher in the HFD + Met group (HFD: 0.821 ± 0.03 vs. HFD + Met: 0.826 ± 0.03; p < 0.001). When the light and dark phases were analyzed separately, the higher RER of the HFD + Met group was only seen in the dark phase, when animals are active and consume most of the food together with metformin (Supplementary Figure 2D). Water intake was significantly increased in the HFD + Met group with 0.11 ± 0.03 ml/day × g body weight vs. 0.08 ± 0.03 ml/day × g body weight in the HFD group (p < 0.01).

Since both groups had the same food intake, we asked if the reduction of weight gain might be a result of malabsorption due to an acute effect of metformin on mitochondrial performance in the intestinal transport epithelium. However, no significant differences were observed in energy content of feces between groups (HFD: 19.35 ± 1.04 kJ/g dry weight vs. HFD + Met: 19.96 ± 0.52, p = 0.23). We also measured total feces production, but found no differences (data not shown).

### 3.3 Continuous metformin application improves glycemic control and lipid metabolism

Intraperitoneal glucose tolerance tests (GTTs) were performed in all groups every two weeks starting at 12 weeks, i.e. 6 weeks after onset of HFD, and blood glucose values during the GTTs done are shown in Supplementary Figure 3. Glucose tolerance was impaired in the HFD groups starting at 14 weeks, resulting in higher glucose levels at each time point compared to normal chow controls. After 18 weeks, glucose tolerance was not different from controls in the HFD + Met group, shown as areas under the curve (Figure 1E). Fasted glucose levels were significantly, but only moderately elevated in the HFD groups (Supplementary Table 1) confirming our previous finding that it takes about 6 months to develop severe T2DM using C57BL6/J mice and this food composition [30].

Metformin did not ameliorate fasting hyperglycemia compared to the HFD group (Supplementary Table 1), probably also because the animals had not ingested the drug for 16 h before blood sampling, and the effects on glucose tolerance seemed rather moderate. However in humans suffering from T2DM, the level of HbA1c is a much more reliable parameter for the long-term follow up of glycemic control [31]. Indeed, HFD significantly increased HbA1c levels, while both metformin treatment schemes completely normalized them, recapitulating its well established effect on glycemic control in humans (Figure 1F and Supplementary Table 1).

Significantly higher levels of cholesterol and triglycerides were seen in the HFD groups compared to controls (Supplementary Table 1), but this was not diminished by metformin treatment. However, HDL levels were significantly higher (176 ± 5 vs. 137 ± 26 mg/dl, p < 0.01), LDL levels were similar (26 ± 4 vs. 23 ± 7 mg/dl, p = 0.4) and VLDL/LDL was significantly lower (0.26 ± 0.04 vs. 0.35 ± 0.07, p < 0.05) in HFD + Met treated animals compared to HFD.

In summary, in mice upon a high fat diet, feeding metformin slows down the development of a type 2 diabetes-like phenotype with profound effects on obesity and long-term glycemic control.

### 3.4 No activation of brown adipose tissue

The increase of energy expenditure could be due to recruitment by metformin of brown adipose tissue (BAT) or of beige fat cells [32] in white adipose tissue depots (WAT). Both would be characterized by increased levels of uncoupling protein 1 (Ucp1) as well as subunits of the mitochondrial respiratory chain [33], here analyzed by the representative core subunit IV of cytochrome c oxidase (CoxIV). However, no differences were found for Cox IV, neither in BAT nor in WAT, and levels of Ucp1 were unchanged in BAT, while it was not detectable at all in WAT (Supplementary Figure 4).

### 3.5 Evidence for a futile glucose–lactate–glucose cycle

**Figure 2:**
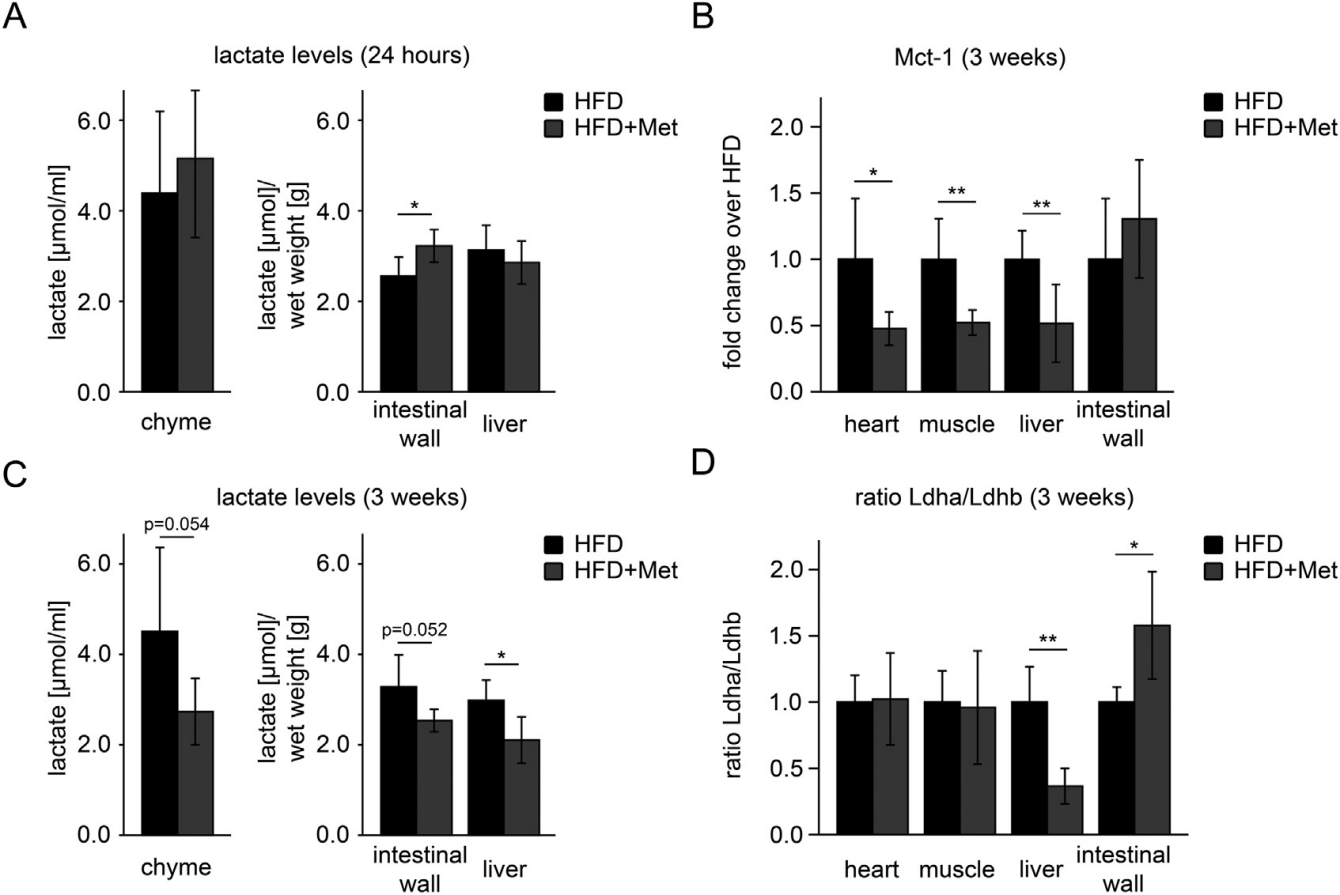
**Modifications of the lactate metabolism in the splanchnic bed in response to metformin treatment**. (A) After 24 h, lactate levels were not different in chyme and liver of mice fed with high fat diet and treated with metformin (HFD + Met; n = 6) and HFD mice (HFD; n = 6; p = n.s.). Lactate levels were significantly higher in the intestinal wall (small intestine and colon, cleared from chyme) of HFD + Met compared to HFD mice (p < 0.05). (B) After 3 weeks of treatment, RT-qPCR results showed significantly reduced expression of monocarboxylate transporter 1 (Mct-1), a transporter specialized for lactate import, in heart (p < 0.05), muscle (p < 0.01), and liver (p < 0.01) and a trend for increased expression in the intestinal wall (p = n.s.) of HFD + Met (n = 5) compared to HFD (n = 6) mice. (C) Lactate levels were clearly reduced in chyme (p = 0.054), intestinal wall (p = 0.052), and liver (p < 0.05) of HFD + Met (n = 5) compared to HFD (n = 6) mice after 3 weeks of treatment. (D) After 3 weeks of treatment, the ratio of lactate dehydrogenase (Ldh) isoforms Ldha/Ldhb were not different in heart and muscle (p = n.s.), significantly decreased in liver (p < 0.01), and significantly increased in intestinal wall (p < 0.05) of HFD + Met (n = 5) compared to HFD (n = 5) mice. Data are expressed as mean ± SD. n indicates the number of individual mice from which chyme and tissues were collected and analyzed. Differences between groups were analyzed by Student's t-test; *p < 0.05 and **p < 0.01.

Metformin has been shown to inhibit complex I of the mitochondrial respiratory chain in hepatocytes in high concentrations (100 μM up to 10 mM; [8]). In general, cells increase lactate production from glucose when mitochondrial ATP production is insufficient or inhibited and indeed, lactate levels rose in the intestinal wall, but not in liver, of mice which had been fed a HFD with metformin for 24 h (Figure 2A). Moreover, in blood taken from the portal vein of these mice, we found a significant increase in lactate and a significant drop of pH as well as of actual base excess (ABE), respectively (Table 1), clearly showing that metformin indeed causes lactic acid production in the intestinal wall, from which it is rapidly removed due to its high perfusion rate. In contrast, neither an increase in lactate nor evidence for acidosis was found in blood taken from the facial or tail vein, respectively (Supplementary Tables 2 and 3), indicating that lactic acid released into the portal circulation does not leave the enterohepatic vascular bed and therefore does not reach the general circulation.

**Table 1:** Increased blood lactate and decreased pH and ABE in blood from the portal vein in response to 24 h of metformin treatment. After 24 h of treatment, blood was analyzed from the portal vein of mice fed with high fat diet and treated with metformin (HFD + Met; n = 5), HFD mice (n = 5) and control mice (n = 6). In detail, pH, partial pressure of carbon dioxide (pCO_2_), partial pressure of oxygen (pO_2_), hemoglobin (Hb), oxygen saturation (sO_2_), hematocrit, potassium, sodium, chloride, hydrogen carbonate (HCO_3_^−^), actual base excess (ABE), glucose, and lactate were measured. Data are expressed as mean ± SD. n indicates the number of individual mice from which blood was collected and analyzed. Differences between groups were analyzed by 1-way ANOVA, followed by Bonferroni's post-hoc test; *p < 0.05 and **p < 0.01 HFD + Met vs. control, ^#^p < 0.05 HFD + Met vs. HFD.

	Control (n = 6)	HFD (n = 5)	HFD + Met (n = 5)
pH	7.08 ± 0.09	7.00 ± 0.06	6.87 ± 0.07**
pCO_2_ [mmHG]	32.8 ± 7.2	29.1 ± 7.3	34.6 ± 5.2
pO_2_ [mmHG]	43.3 ± 16.8	41.0 ± 6.6	44.3 ± 12.3
Hb [g/dl]	6.9 ± 1.7	6.0 ± 1.8	6.5 ± 1.2
sO_2_ [%]	51.9 ± 20.9	46.0 ± 11.9	37.5 ± 11.2
Hematocrit [%]	21.6 ± 5.1	18.8 ± 5.5	20.5 ± 3.6
Potassium [mmol/l]	1.7 ± 0.5	1.4 ± 0.4	1.8 ± 0.4
Sodium [mmol/l]	138.6 ± 4.6	136.0 ± 3.4	137.4 ± 2.1
Chloride [mmol/l]	85.3 ± 8.6	83.6 ± 6.9	93.6 ± 8.3
HCO_3_^−^ [mmol/l]	9.5 ± 3.0	7.0 ± 2.6	6.2 ± 1.5
ABE [mmol/l]	−18.8 ± 3.9	−22.1 ± 3.0	−24.6 ± 1.8*
Glucose [mmol/l]	6.5 ± 2.4	6.4 ± 2.5	9.4 ± 2.7
Lactate [mmol/l]	2.8 ± 0.5	2.5 ± 0.8	3.9 ± 0.7 *^#^

**Figure 3:**
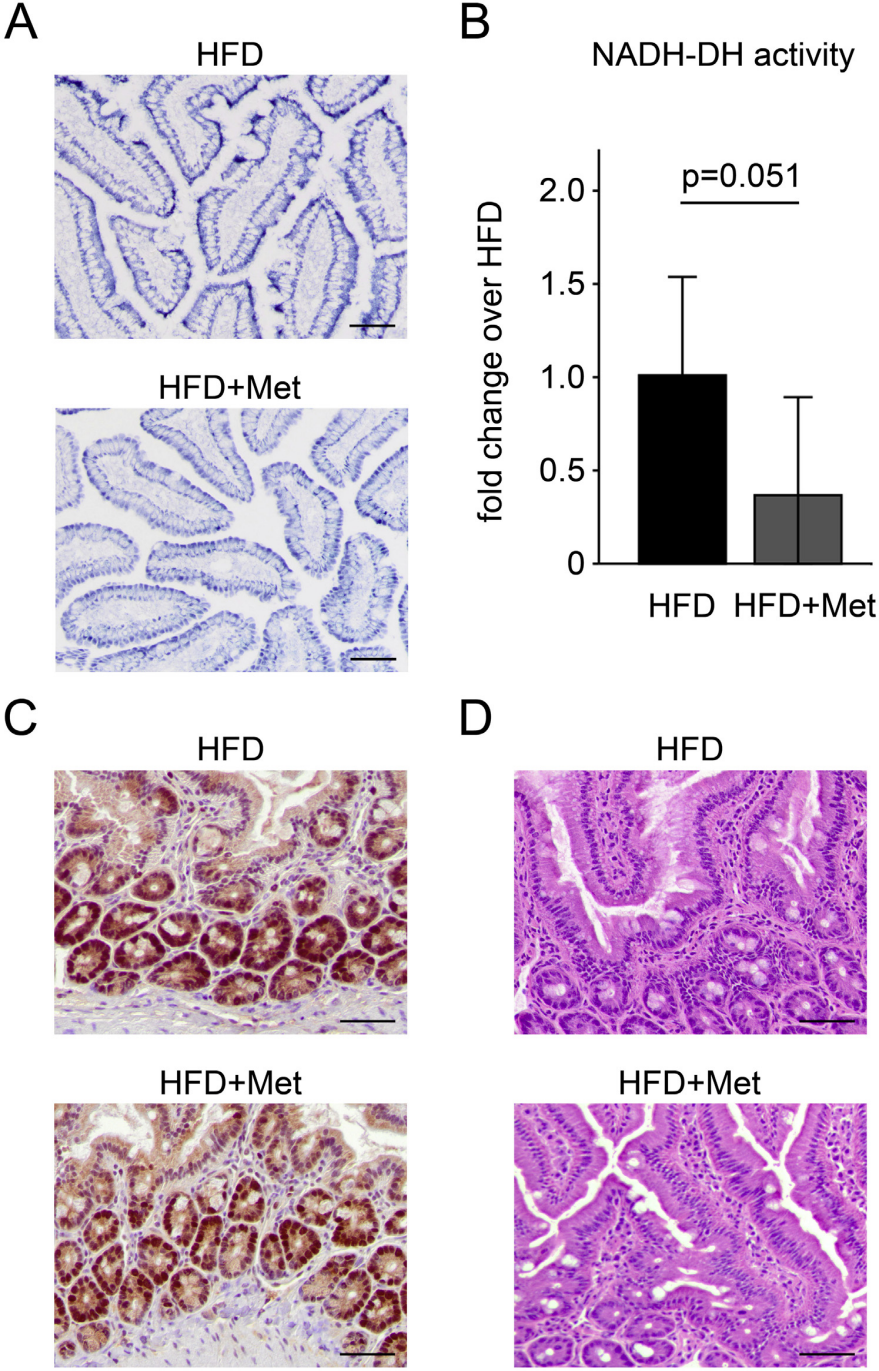
**Metformin acted as a mitochondrial inhibitor in the intestinal wall**. (A) Staining for mitochondrial complex I (NADH-DH) activity in the intestinal wall (duodenum) and (B) its quantification showed about 3fold lower complex I (NADH-DH) activity in mice fed with high fat diet and treated with metformin (HFD + Met) compared to HFD mice (2–4 samples from n = 2; p = 0.051). (C) There were no differences in the amount of proliferating cells, visualized by PCNA staining, and (D) no signs of tissue damage, visualized by H&E staining, in the intestinal wall of HFD + Met compared to HFD mice. Data are expressed as mean ± SD. n indicates the number of individual mice from which intestinal wall was collected and analyzed. Differences between groups were analyzed by Student's t-test; p = 0.05. Scale bars: 50 μm.

Since our results show that metformin indeed acts as a mitochondrial inhibitor in the wall of the small intestine, we stained samples of the duodenum for NADH-“diaphorase” activity, which is due to mitochondrial complex I activity in cells rich in mitochondria. This demonstrated the high abundance of this complex in the intestinal epithelium, both apical as well as basal (Figure 3A), and showed that complex I activity was about 3 fold lower in metformin consuming mice (Figure 3B). Staining for proliferating cells (PCNA; Figure 3C) and with H&E (Figure 3D) showed no difference, demonstrating that metformin does not cause any tissue damage at the administered dose.

Our results thus strongly suggest conversion of glucose to lactate in the wall of the small intestine in the presence of metformin due to complex I inhibition and the conversion of lactate back to glucose in the liver. Indeed, glycogen levels were considerably lower in livers of mice after 12 weeks of HFD + Met, further corroborating this hypothesis (control: 355 ± 55; HFD: 301 ± 29 vs. HFD + Met: 137 ± 39 μMol glycosyl units/g wet weight, p < 0.01 vs. HFD and vs. control, respectively).

**Figure 4:**
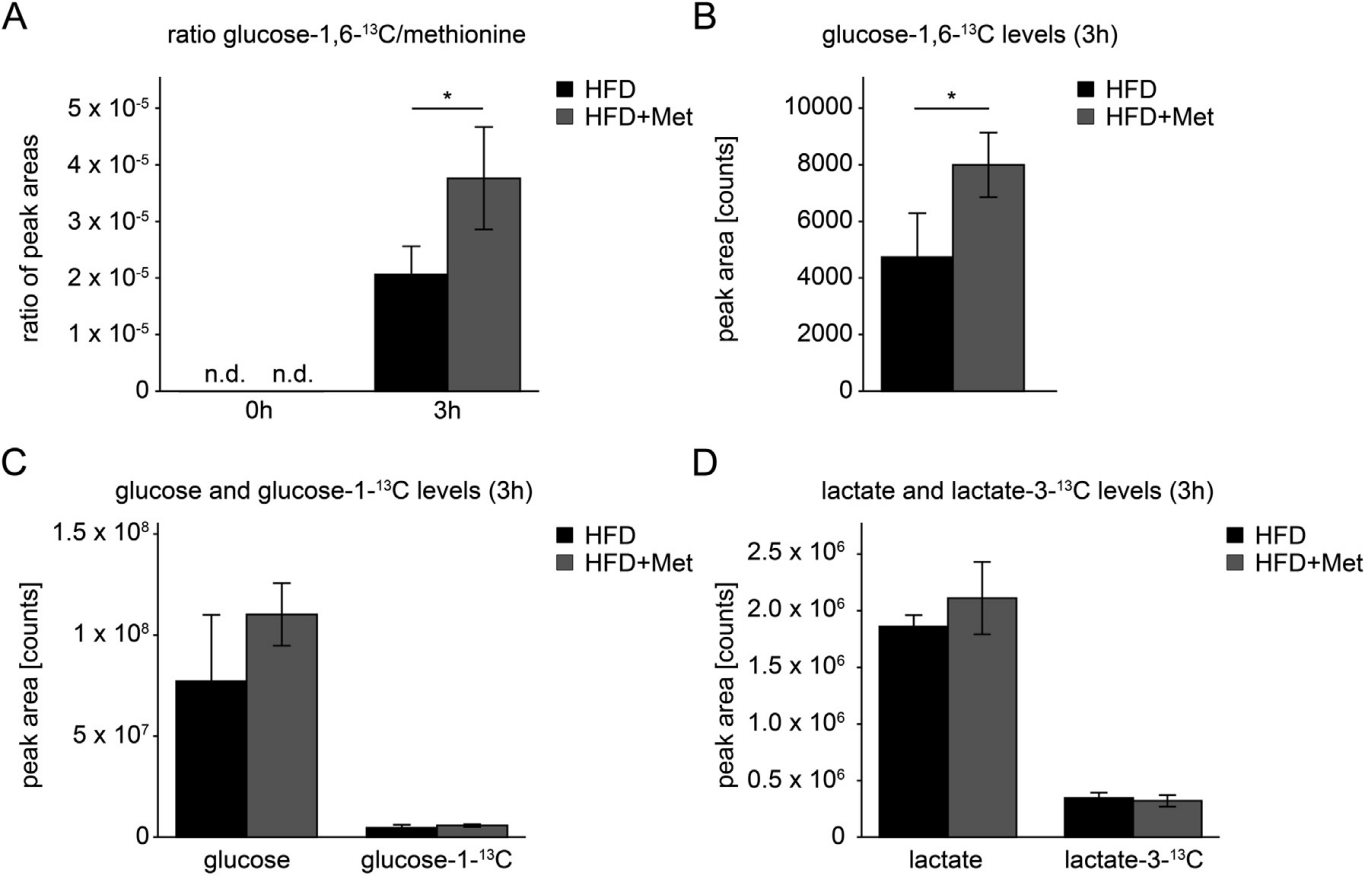
**Induction of glucose-1-^13^C to glucose-1,6-^13^C conversion by metformin treatment**. (A) At baseline (0 h), doubly labeled glucose-1,6-^13^C was not detectable (n.d.) in peripheral blood, collected by cheek punch, and after 3 h of feeding; the ratio of doubly labeled glucose-1,6-^13^C to methionine was significantly higher in mice fed with high fat diet and treated with metformin (HFD + Met; n = 4) compared to HFD mice (n = 3; p < 0.05). (B) After 3 h of feeding, doubly-labeled glucose-1,6-^13^C was significantly higher in sera, collected from the left ventricle, of HFD + Met (n = 4) compared to HFD mice (n = 3; p < 0.05). (C) After 3 h of feeding, the amount of glucose and glucose-1-^13^C and (D) lactate and lactate-3-^13^C was not different in sera, collected from the left ventricle, of HFD + Met (n = 4) compared to HFD mice (n = 3; p = n.s.). Note, that because of variable blood sample size, the cheek punch data needed to be normalized, while data for serum generated from ventricular blood show absolute peak areas. Data are expressed as mean ± SD. n indicates the number of individual mice from which blood was collected and analyzed. Differences between groups were analyzed by Student's t-test; *p < 0.05.

In order to demonstrate this pathway directly, mice were given labeled glucose-1-^13^C in HFD. Since the natural abundance of the ^13^C-isotope is only 1.1%, no doubly labeled glucose-1,6-^13^C was detected in the blood samples before the experiment (Figure 4A and Supplementary Figure 6). After 3 h of feeding, newly generated, doubly labeled glucose-1,6-^13^C was nearly two-fold higher in the presence of metformin compared to controls (Figure 4A,B). Glucose-^13^C, lactate-^13^C, unlabeled glucose, and lactate were not significantly altered in peripheral blood (Figure 4C,D).

To even further support our findings (Figure 6), the expression of the monocarboxylate transporter isoform 1 as well as lactate dehydrogenase isoforms was analyzed by RT-qPCR in mice after 3 weeks of HFD, i.e. when we expected that processes adaptive to the constant release of lactate from the intestine had been fully initiated. In liver, heart and muscle, expression of the monocarboxylate transporter Mct-1 (*Slc16a1*), which is specialized for lactate import, was significantly decreased (Figure 2B), while there was a trend for increased expression in the intestinal wall, in line with improved extraction of lactate from the chyme at that later stage (Figure 2C). In addition, the ratio of lactate dehydrogenase (Ldh) isoforms Ldha/Ldhb was increased in the intestinal wall, favoring the conversion of pyruvate to lactate, while it was decreased in the liver, favoring the opposite reaction (Figure 2D). Indeed, liver tissue showed significantly lower levels of lactate at this stage (Figure 2C).

**Figure 5:**
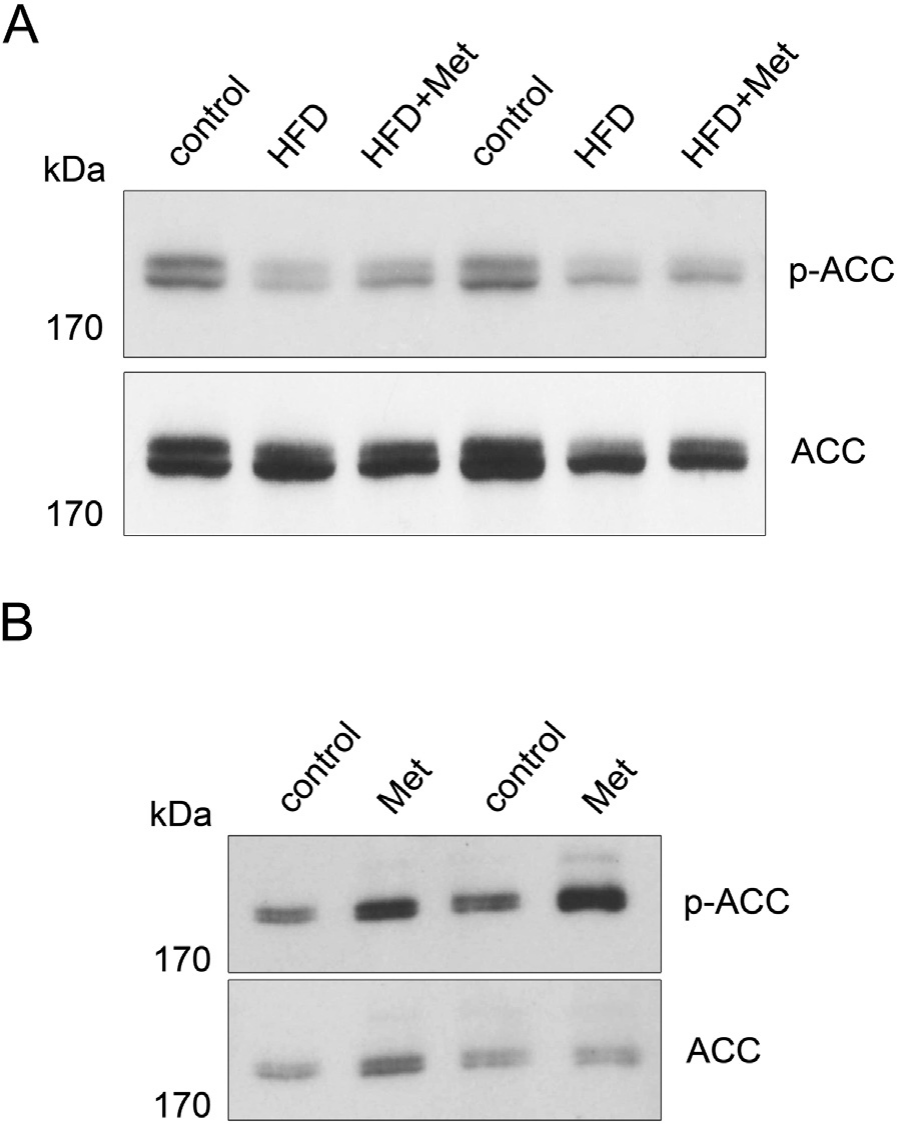
**Continuous treatment with metformin did not enhance phosphorylation of ACC at Ser79 in liver**. (A) After 12 weeks of treatment, phosphorylation of acetyl-CoA carboxylase at Ser79 (p-ACC) was not different in livers of mice fed with high fat diet and treated with 500 mg metformin kg^−1^ per day (HFD + Met), HFD mice (HFD) and control animals. Expression of unphosphorylated acetyl-CoA carboxylase (ACC) was comparable between all three groups. (B) However, 3 h after giving 500 mg metformin kg^−1^ in one bolus by gavage to mice (Met), phosphorylation of ACC at Ser79 was clearly induced in livers compared to livers from non-treated control mice. Expression of ACC was comparable between both groups.

**Figure 6:**
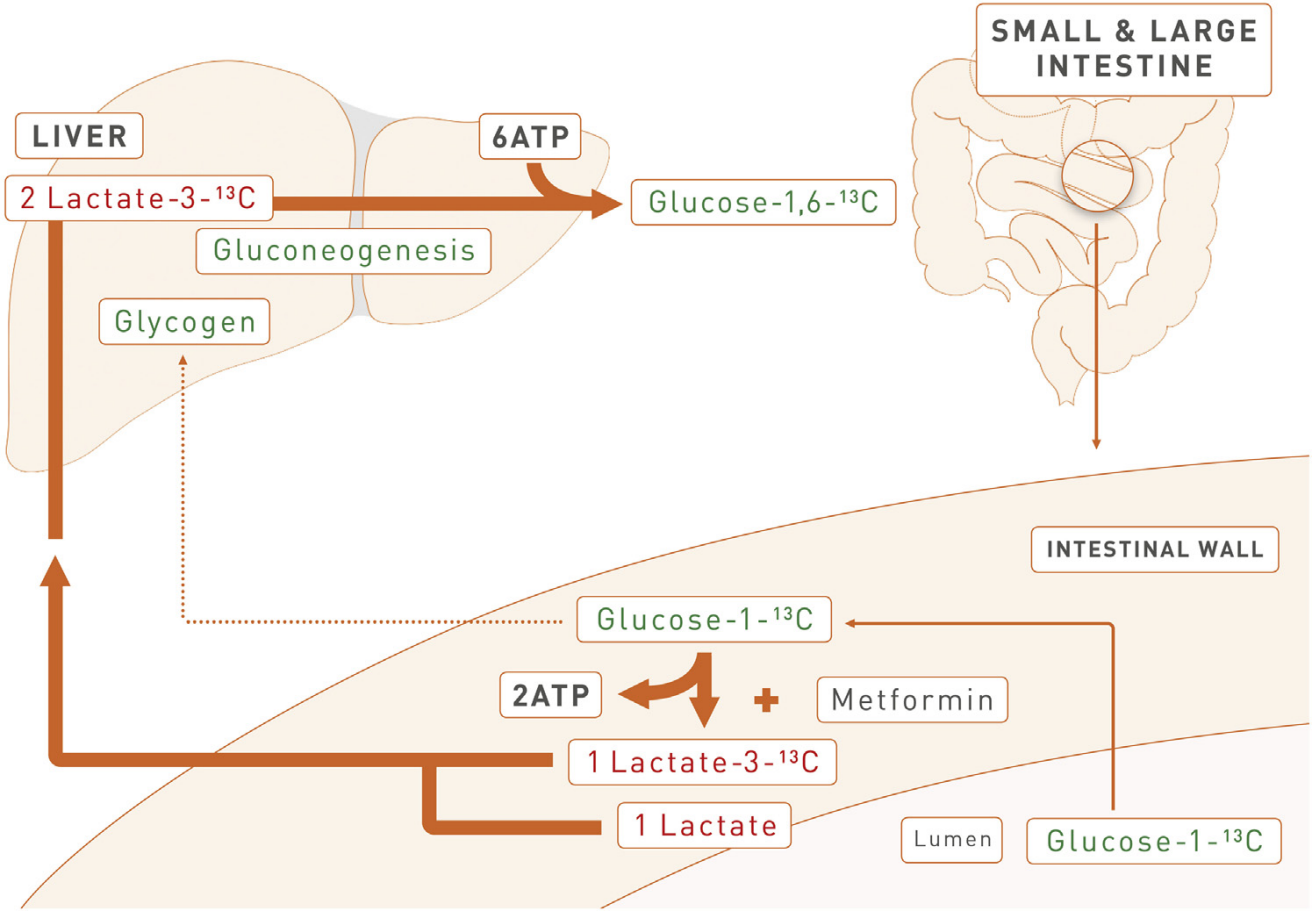
**Scheme illustrating the energy consuming, futile glucose–lactate–glucose cycle in the splanchnic bed induced by metformin in the intestine**.

Our results seem to be in conflict with a large body of literature showing inhibition of hepatic glucose production by metformin, although in the long term, we also found a significant improvement of glycemic control (Figure 1E). Therefore, we analyzed phosphorylation of ACC at Ser 79, a well-known readout of metformin action in liver, in mice after 12 weeks of treatment, but found no significant changes compared to controls (Figure 5A). However, applying metformin at a single high dose by gavage indeed led to increased ACC phosphorylation (Figure 5B), as shown by others who used daily i.p. injections or gavage [16], [34]. This demonstrates that at our feeding conditions, metformin obviously did not reach the liver in concentrations high enough to activate ACC phosphorylation and other downstream pathways. In order to substantiate this, metformin concentrations were analyzed and found to be 13 ± 5, 40 ± 13 and 37 ± 27 μmol/L after feeding HFD with 0.5%, 1% and 2.5% metformin, respectively. Thus, our feeding regime with 0.5% metformin resulted in levels which are in the lower range of concentrations reached by others in animals and humans (10–40 μmol/L) [34].

## 4 Discussion

Numbers of prescriptions of metformin grew constantly in the last 20 years and it is now estimated to be given to at least 150 million people suffering from T2DM worldwide [34]. Since high blood glucose causes the most severe complications in T2DM, researchers interested in understanding its mode of action mostly concentrated on the suppression of glucose production in the liver, which certainly occurs *in vivo* in patients upon treatment [35].

In contrast, surprisingly little attention has been paid to the mechanisms responsible for lowering body weight upon or maintaining body weight during metformin treatment [4], [5], [28], [29], [35], [36], [37]. This effect cannot be explained by metformin's suppressive effect on hepatic glucose output, since gluconeogenesis is an energy consuming process; thus, metformin should decrease energy expenditure and therefore increase body weight. However, weight loss alone is a powerful means to improve glucose homeostasis in T2DM; therefore, we felt that deciphering the responsible mechanism also deserves attention. Indeed, in our study metformin not only slowed down the development of a type 2 diabetes-like phenotype with disruption of glycemic control and dyslipidemia, as expected, it also significantly slowed down weight gain. We first excluded the most trivial reasons like decreased food intake due to metformin's bitter taste, increased locomotion due to enhanced foraging activity, or malabsorption due to inhibition of active transport processes in the intestine. We also excluded activation of BAT or recruitment of beige adipocytes in WAT, respectively.

Instead, the slower weight gain can be explained by the marked increase in energy expenditure we found after metformin treatment. We reasoned that metformin is an inhibitor of complex I of the respiratory chain, at least at high concentrations [7], [38], which are certainly reached in the intestine after oral administration [18]. It has been reported before that metformin causes increased conversion of glucose to lactate in the intestinal wall, when given as single bolus at a dose about five times higher than the maximum daily dose given to patients ([39], summarized in [19]). This treatment did not cause malabsorption of glucose, but the local lactic acidosis may explain the gastrointestinal disturbances reported for about 20–30% of patients taking metformin [11], [40], [41], some of which can even be associated with polymorphisms of putative metformin transporters [41]. Indeed, we found inhibition of complex I in the intestinal epithelium, followed by increased lactate levels and accompanied by a drop in pH in portal vein blood, but not in peripheral venous blood, and therefore conclude that lactic acid is converted back to glucose in the liver (Figure 6). Most convincingly, we demonstrate that circulating levels of doubly labeled glucose-1,6-^13^C were twice as high in the presence of metformin after oral administration of glucose-1-^13^C (Figure 4A,B). Breakdown of glucose-1-^13^C will yield one molecule of lactate-3-^13^C, which can be converted in the aldolase reaction into doubly-labeled glucose-1,6-^13^C, when by chance two labeled lactate-3-^13^C molecules are used for gluconeogenesis (Figure 6). Since more lactate-3-^13^C is generated in the intestinal wall in the presence of metformin (Figure 2A) and released into the portal vein (Table 1), it is more likely that doubly labeled glucose-1,6-^13^C molecules are generated in the liver.

Eventually, this process consumes 6 mol of ATP, when 1 mol of glucose is produced from 2 mol of lactate [42], which had generated 2 mol of ATP in the intestinal wall when derived from glucose, either taken from the lumen or from blood. We propose that this futile cycle, together with energy consuming mechanisms maintaining pH-homeostasis, largely explains the increased energy expenditure and, consequently, the reduced weight gain of metformin treated mice under a high fat diet.

Indeed, liver glycogen content was significantly lower after metformin treatment, since additional resources are needed in the liver to support the energy consuming glucose production (Figure 6, dotted line). In addition, our model is reinforced by the observation that after 3 weeks, several adaptive processes have occurred. In the intestinal chyme, the level of lactate was significantly lower in the metformin treated group, showing that the capacity to extract lactate from chyme has increased (Figure 2C), probably by augmented expression of the responsible inward transporter Mct-1 ([43]; Figure 2B; although not significant), and by a change in the ratio of Ldh isoforms [44] favoring production of lactate from pyruvate derived from absorbed or also blood-born glucose (Figure 2D). Other metabolic organs have down-regulated Mct-1 (Figure 2B), probably as a protection against inflow of lactate together with the accompanying proton. This may help to avoid intracellular acidosis in these organs when intestinal levels of metformin are high and lactic acid does appear in the periphery. In liver, the ratio of Ldh isoforms has changed favoring production of pyruvate from lactate (Figure 2D) as a substrate for gluconeogenesis. Additional data supporting our model are an increased respiratory exchange ratio (RER) during the dark phase, when animals consume most of the drug (Supplementary Figure 3D), which is a well-established indicator of lactic acidosis, and a significantly increased water intake under metformin, which may be due to increased water loss by hyperventilation as a compensation for lactic acidosis.

Although we saw long-term improvement of glycemic control, based on HbA1c measurements and GTTs, we did not observe lower blood glucose in the fasted state, indicating that metformin did not importantly suppress hepatic gluconeogenesis in our mice. This seems to contradict an immense body of literature, both in patients [35] as well as in mice [12], [13]. However, since phosphorylation of ACC was unchanged (Figure 5A), we conclude that under the conditions of our experiment, metformin did not reach the liver in concentrations high enough to induce this downstream effect well described in previous studies. Indeed, 13 ± 5 μmol/L metformin which we obtained in peripheral blood with our feeding regime of 0.5% metformin is in the lowest range of concentrations reached by bolus administration in animals and humans reported by others [34]. When we administered the daily dose of metformin by gavage, as done by others, we also found increased ACC phosphorylation (Figure 5B), emphasizing that hepatic metformin action is highly dose dependent, as discussed recently [34]. Patients take metformin (500–1000 mg) two or three times a day together with meals [39], and mice, in which phosphorylation of ACC has been shown in liver, are routinely given the drug by bolus gavage (15–500 mg/kg [12], [45], [46]) or daily i.p. injection (150 mg kg^−1^ [16]). In contrast, our mice consume 500 mg kg^−1^ distributed over 24 h, mostly, but not exclusively, during the night, thus other mechanisms due to different kinetics had to be expected.

Metformin has been shown to activate anaerobic glycolysis in isolated intestinal preparations already at concentrations of 10 μM [47], probably due to the efficient translocation into enterocytes mediated by the apical transporters PMAT (*SLC29A4*) and SERT (*SLC6A4*) [48], [49], but concentrations of 100 μM to even 10 mM have to be used in hepatocytes to inhibit mitochondrial respiration there [38]. This is much higher than therapeutic concentrations and was definitely not reached in our setting.

Therefore, we propose that the energy-consuming, futile glucose–lactate–glucose cycle, for which we have provided strong evidence here, will also operate in patients as long as the metformin concentration in the intestinal epithelium is high and low in the liver. Lactic acidosis remains mostly confined to the enterohepatic circulation, but may also affect other organs, which can be compensated by adaptive processes, e.g. changes of Mct-1 and Ldh isoform expression, as shown here in mice. The very rare cases in which patients experience systemic lactic acidosis are due to exceptionally high levels of metformin, e.g. because of inadequate excretion in patients with renal dysfunction [50], [51] or polymorphisms in the organic cation transporters responsible for its route [41].

In patients taking their daily metformin dose, we propose two consecutive steps, as metformin will be first transported into the intestinal epithelium, which serves as a sink and where it inhibits the mitochondrial respiratory chain. It has been clearly shown that the drug is only slowly released into the portal vein [39], where it will inhibit gluconeogenesis in the liver only if concentrations are sufficiently high, which seems certainly to be the case in patients under normal treatment regimens [35]. Formulations of metformin with retarded release are already available on the market, and these may be adjusted to contain even higher concentrations in order to effectively drive both effects, increased energy expenditure due to the futile cycle described here as well as inhibition of gluconeogenesis, which will both ameliorate type 2 diabetes. Indeed, our work is strongly supported by a recent paper showing in large patient cohorts that even the glucose lowering effect of the drug may reside in the gut if given as a delayed-release formulation (MetDR) [52]. To what extent the recently described effects of metformin on the composition of the gut microbiota contributes to our findings is unclear. However, it has been reported that in patients taking metformin, that there is a shift to microbial species producing butyrate and propionate, metabolites which, in turn, stimulate intestinal gluconeogenesis [53], which in rodents results in a beneficial effect on glucose and energy homeostasis with reductions in hepatic glucose production, appetite and body weight [54] (reviewed in [55]).

## Author contributions

The authors contributed to this work in the following ways: P.S., A.T., and I.B.-M., performed conception and design, experiments, data analysis and interpretation, and manuscript writing; M.G., A.R.K., J.R., and J.A. performed experiments, data analysis, and interpretation; M.K., M.H.A., D.G., and A.S.-K. performed conception and design, financial support, data analysis, and interpretation; and R.J.W. performed conception and design, financial support, data analysis and interpretation, manuscript writing, and final approval of manuscript.
