# Supplementary material for: Metformin causes a futile intestinal–hepatic cycle which increases energy expenditure and slows down development of a type 2 diabetes-like state
Source: Mol Metab. 2017 May 5;6(7):737–47. doi: 10.1016/j.molmet.2017.05.002 (PMC5485244; doi:10.1016/j.molmet.2017.05.002)
Supplement: Supplementary file 1 [file mmc1.docx]

**Metformin causes a futile intestinal-hepatic cycle which increases energy expenditure and slows down development of a type 2 diabetes-like state**

Philipp Schommers^1,2^*, Anna Thurau^1^*, Insa Bultmann-Mellin^3^*, Maria Guschlbauer^3^, Andreas R. Klatt^4^, Jan Rozman^5,6^, Martin Klingenspor^5,6,7,8^, Martin Hrabe de Angelis^5,6^, Jens Alber^9^, Dirk Gründemann^10^, Anja Sterner-Kock^3^ and Rudolf J. Wiesner^1,11,12^

^1^Center for Physiology and Pathophysiology, Institute for Vegetative Physiology, Medical Faculty, University of Köln, 50931 Köln, Germany

^2^Department I of Internal Medicine, Medical Faculty, University of Köln, 50931 Köln, Germany

^3^Center for Experimental Medicine, Medical Faculty, University of Köln, 50931 Köln, Germany

^4^Institute for Clinical Chemistry, Medical Faculty, University of Köln, 50931 Köln, Germany

^5^German Mouse Clinic, Helmholtz Zentrum München, German Research Center for Environmental Health, 85764 Neuherberg, Germany

^6^German Center for Diabetes Research (DZD), 85764 Neuherberg, Germany

^7^Chair of Molecular Nutritional Medicine, Technische Universität München, Else Kröner-Fresenius Center for Nutritional Medicine, 85350 Freising, Germany

^8^ZIEL – Institute for Food and Health, Technische Universität München, 85350 Freising

^9^Max-Planck Institute for Metabolism Research, 50931 Köln, Germany

^10^Department of Pharmacology, Medical Faculty, University of Köln, 50931 Köln, Germany.

^11^Center for Molecular Medicine Cologne (CMMC), University of Köln, 50931 Köln, Germany

^12^Cologne Excellence Cluster on Cellular Stress Responses in Aging-associated Diseases (CECAD), University of Köln, 50931 Köln, Germany

*Authors contributed equally

Corresponding author:

Rudolf J. Wiesner

Center for Physiology and Pathophysiology, University of Köln,

Robert-Koch-Str. 39

50931 Köln, Germany.

Tel.: +49 221 478 3610

Fax: +49 221 478 6965

rudolf.wiesner@uni-koeln.de

## Supplementary Materials and Methods

Animal cohorts

In an initial experiment, mice were fed a HFD starting at 3 weeks of age. Intraperitoneal glucose tolerance tests (GTT) were performed every 2 weeks for another 18 weeks to follow the development of glucose intolerance, which occurred after 6 weeks of HFD (data not shown). According to these results, in a large cohort, animals were separated into four different groups at the age of 6 weeks (n=8; Supplementary Figure 1). Controls received standard chow. The HFD+lateMet group, received HFD+0,5% of metformin at 12 weeks of age, when animals started to show glucose intolerance. The HFD+Met group was given HFD+0,5% of metformin at 6 weeks of age, resulting in 6 weeks longer metformin treatment compared to the HFD+lateMet group. A third cohort of animals was given HFD and HFD+Met, respectively (n=6), at the age of 6 weeks and were sacrificed 3 weeks later. Here, liver, heart, M. gastrocnemius, small intestinal wall and intestinal chyme were rapidly frozen for lactate measurements as well as to study the adaptations of organs to treatment. For PhenoMaster experiments, a fourth cohort of mice was used that were treated with either HFD or HFD+met (n=16 each, males and females) from the age of 6 weeks on the age of 6 weeks for 12 weeks. At the age of 18 weeks mice were kept in an open circuit measurement system (PhenoMaster, TSE Systems GmbH, Bad Homburg, Germany) for 48 hours.

To obtain data on the short term action of the drug, a fifth cohort of animals were fasted overnight for 16 hours, were given access to normal chow, HFD or HFD+Met, respectively, the next morning, and were sacrificed 24 hours later under deep anaesthesia. Blood was taken from the portal vein (V. portae hepatis), the tail vein (V. caudalis mediana) or the cheek pouch (V. temporalis superficialis) and blood gas analysis was performed using an ABL800 Flex blood gas analyzer (Radiometer, Willich, Germany). Liver, small intestinal wall and intestinal chyme were frozen for lactate measurements. To obtain data on the well-known hepatic action of the drug, in a sixth cohort 500 mg metformin/kg body weight was given in one bolus by gavage to mice fasted overnight for 16 hours. Animals were sacrificed 3 hrs later and livers were quickly frozen in liquid nitrogen.

To investigate glucose metabolism in the splanchnic bed, in a seventh cohort animals were fasted overnight for 16 hours and then were given free access to a HFD (see above) in which the 19% disaccharide had been replaced by fiber, in which was 1 g of glucose-1-^13^C (Sigma Aldrich, Darmstadt, Germany, # 297046) per 10 g of food was supplemented and to which 0.5% metformin was added for one group. After 3 hrs, blood was taken from the cheek pouch, animals were sacrificed and serum was prepared from blood taken from the ventricular cavity.

HE and PCNA staining procedures

Sections of 2-3 μm were also stained with haematoxylin-eosin according to standard protocols. For proliferating cell nuclear antigen (PCNA) staining, cryopreserved tissue were fixed in 4% paraformaldehyde and embedded in paraffin. 4-μm sections were prepared and deparaffinized. Antigen retrieval was performed in 10 mM citrate puffer (pH 6.0) for 20 minutes in a microwave followed by treatment with 3% H_2_O_2_ for 15 minutes at room temperature. Incubation with PCNA primary antibody (1:6,000, Abcam, Cambridge, UK, #ab2426) in dilution buffer was performed for 1 hour at room temperature. For detection, Supervision 2-Single Species HRP Polymer (DCS Innovative Diagnostik-Systeme, Hamburg, Germany) and Permanet AEC-Kit (ZYTOMED Systems, Berlin, Germany) were used according to manufacturers’ protocols. Sections incubated without primary antibody were used as controls. Images were taken using an Olympus BX-40 microscope (Olympus, Hamburg, Germany).

Determination of pACC/ACC ratios

Proteins from liver samples after 12 weeks of treatment were lysed in RIPA buffer (50 mM Tris, 1% NP-40, 0.25% sodium deoxycholate, 150 mM NaCl, 2mM EDTA), separated by 8 % SDS-gel electrophoresis and blotted onto a nitrocellulose membrane (Schleicher & Schuell BioScience, Dassel, Germany). A cocktail of protease and phosphatase inhibitors was freshly added to the RIPA buffer before use (Roche Diagnostics, Mannheim, Germany). Membranes were blocked with 3% skimmed-milk in PBS and incubated with primary antibodies raised against: pACC 1:1,000 (rabbit anti-phosphoACC (Ser79), Cat. #3661) and 1:1,000 ACC (rabbit anti-ACC, Cat. #3676, all antibodies from Cell Signaling Technology, Leiden, The Nederlands) and peroxidase-conjugated secondary donkey anti-rabbit antibody (1:5,000; Jackson ImmunoResearch, Newmarket, Suffolk, UK). Signals were detected using the enhanced chemiluminescence Western blotting detection reagent (ECL, Perkin Elmer, Rodgau, Germany).

Expression of monocarboxylate transporters and lactate dehydrogenase isoforms

Total RNA was isolated from target tissues with TRIzol reagent (PEQLAB Biotechnologie, Erlangen, Germany) according to manufacturer’s instruction. Reverse transcription was performed with oligo dT primers and SuperScript III Reverse Transcriptase (Life Technologies, Darmstadt, Germany) using 1 µg of total RNA according to manufacturer’s instructions. qPCR was done using the PlatinumH Quantitative PCR SuperMix-UDG w/ROX kit (Life Technologies, Darmstadt, Germany). The primers used are provided in the Table below. Relative mRNA content in samples was normalized to mRNAs for glyceraldehyde-3-phosphate dehydrogenase PCR products were verified by agarose gel electrophoresis, triplicate reactions were set up and qPCR was performed using the 7500 Real Time PCR System (Life Technologies, Darmstadt, Germany). Calculations were performed by a comparative 2^-ΔΔC^_T_ method [1].

Quantification of Ucp1 expression in BAT and WAT

Frozen subcutaneous white adipose tissue (WAT) and interscapular brown adipose tissue (BAT) from animals after 12 weeks of HFD or HFD+Met was crushed in liquid nitrogen using a mortar and pestle. Tissue aliquots were homogenized in RIPA buffer (see above). Following homogenization, extracts were centrifuged at 16,000xg, 4°C for 15 min and clear supernatants were collected. Protein concentrations were determined using bicinchoninic acid and bovine serum albumin for calibration. For gel electrophoresis (12.5% SDS-PAGE), 30 µg of protein were denatured in 2 x SDS sample buffer for 5 min at 95°C. As a reference, different amounts of recombinant murine Ucp1 (mUcp1) were denatured (7.8 – 500 ng). All samples were separated by gel electrophoresis and blotted by semi-dry transfer for 1.5 h at 1 mA/cm^2^ to a nitrocellulose membrane. Membranes were blocked in TBS with 3% BSA at 4°C. All antibodies were diluted in TBST with 1:10,000 for Ucp1 (rabbit anti-hamster UCP1 [2] 1:5,000 for CoxIV (rabbit anti-human COXIV, Cell Signaling Technology, Leiden, The Nederlands, #4844) and 1:5,000 for a-actin (rabbit anti-chicken actin, clone C4, Cat. #MAB1501, Merck, Millipore, Billerica, USA). The infrared dye labelled secondary antibodies (IRDye® 600CW and IRDye® 800CW, LI-COR Bioscience, Lincoln, USA) were diluted 1:20,000, respectively. Detection and signal quantitation was performed using an infrared scanner and the corresponding software analysis tools (Odyssey, LI-COR Bioscience, Lincoln, USA).

Hba1c, blood lipids and lipoprotein levels and liver glycogen

Blood was taken from the heart directly after sacrificing the animals at 18 weeks of age. Glycated hemoglobin A1c (HbA1c) levels were measured in whole blood samples using two methods. First the **SEBIA CAPILLARYS HbA1c kit was** used on a CAPILLARYS 2 Flex Piercing analyzer (Sebia, Norcross, USA) and hemoglobin and hemoglobin variants (e.g. HbA1c) were detected at 415nm. Second, HbA1c levels were measured by a turbidimetric inhibition immunoassay using a Tina-Quant HbA1c second generation assay (Roche Diagnostics, Mannheim, Germany). Both tests were performed and evaluated according to manufacturer’s instructions.

Cholesterol, triglyceride, HDL and LDL levels were determined in serum by the enzymatic CHOD-PAP, GPO-PAP, HDLC3, and LDL-C assays, respectively, on a Modular P800 analyzer (Roche Diagnostics, Mannheim, Germany) according to manufacturer’s instructions.

Serum lipoproteins were separated with Hydragel LDL/HDL/CHOL direct kits on a semi-automated agarose gel electrophoresis system (Hydrasis, Sebia, Norcross, USA). The electrophoretic patterns were evaluated by densitometry using the Hyrys 2 Densitometer (Sebia, Norcross, USA) according to the manufacturer’s instructions.

Glycogen content was measured after precipitation from liver samples of animals at 18 weeks of age, acid hydrolysis followed by neutralization and determination of liberated glucose [3].

Feces analysis

A possible inhibition of gastrointestinal functions by metformin was investigated by determining energy content of feces using Fourier-transformed infrared (FT/IR) reflectometry of dried, homogenized feces boli samples, collected from single caged mice during the indirect calorimetry trial. Triplicates of samples of individual mice (ca. 50 mg each) were analyzed based on diffuse reflection using a Tensor 27 (Bruker Optics, Vienna, Austria). Prior to the FT/IR measurement samples were stored in a drying oven for one week at 63°C with open lid for complete desiccation. Material was then homogenized to a fine powder in a mixer mill (model MM400, Retsch, Haan, Germany) with 3 steel balls (7mm diameter) for 1:45 min at a frequency of 30 Hz. The homogenized material was evenly loaded on a 96-well microplate for reflectometry. Single spectra were averaged from 60 scans. First derivation transformation of single spectra was then conducted before averaging spectral information. We developed a calibration model to estimate energy content [kJ g^-1^ dry mass] of dried feces samples from FT/IR spectra with bomb calorimetry as the reference method. In brief, we determined energy content of 460 samples by bomb calorimetry and conducted FT/IR reflectometry in triplicate aliquots as described above. The calibration model was calculated using FTIR Quant 2 (Bruker Optics, Vienna, Austria) and validated by independent analysis of energy content by bomb calorimetry and FT/IR reflectometry in parallel (observed vs. predicted energy content: y = 0.8022 * x +3215.9, n = 60, r^2^=0.881).

## Supplementary References

[1] Livak KJ and Schmittgen TD, Analysis of relative gene expression data using real-time quantitative PCR and the 2(-Delta Delta C(T)) Method. Methods, 2001;25:402-8.

[2] Klingenspor M, Ebbinghaus C, Hulshorst G, Stohr S, Spiegelhalter F, Haas K, et al., Multiple regulatory steps are involved in the control of lipoprotein lipase activity in brown adipose tissue. J Lipid Res, 1996;37:1685-95.

[3] Crosson SM, Khan A, Printen J, Pessin JE and Saltiel AR, PTG gene deletion causes impaired glycogen synthesis and developmental insulin resistance. J Clin Invest, 2003;111:1423-32.

## Supplementary Figures


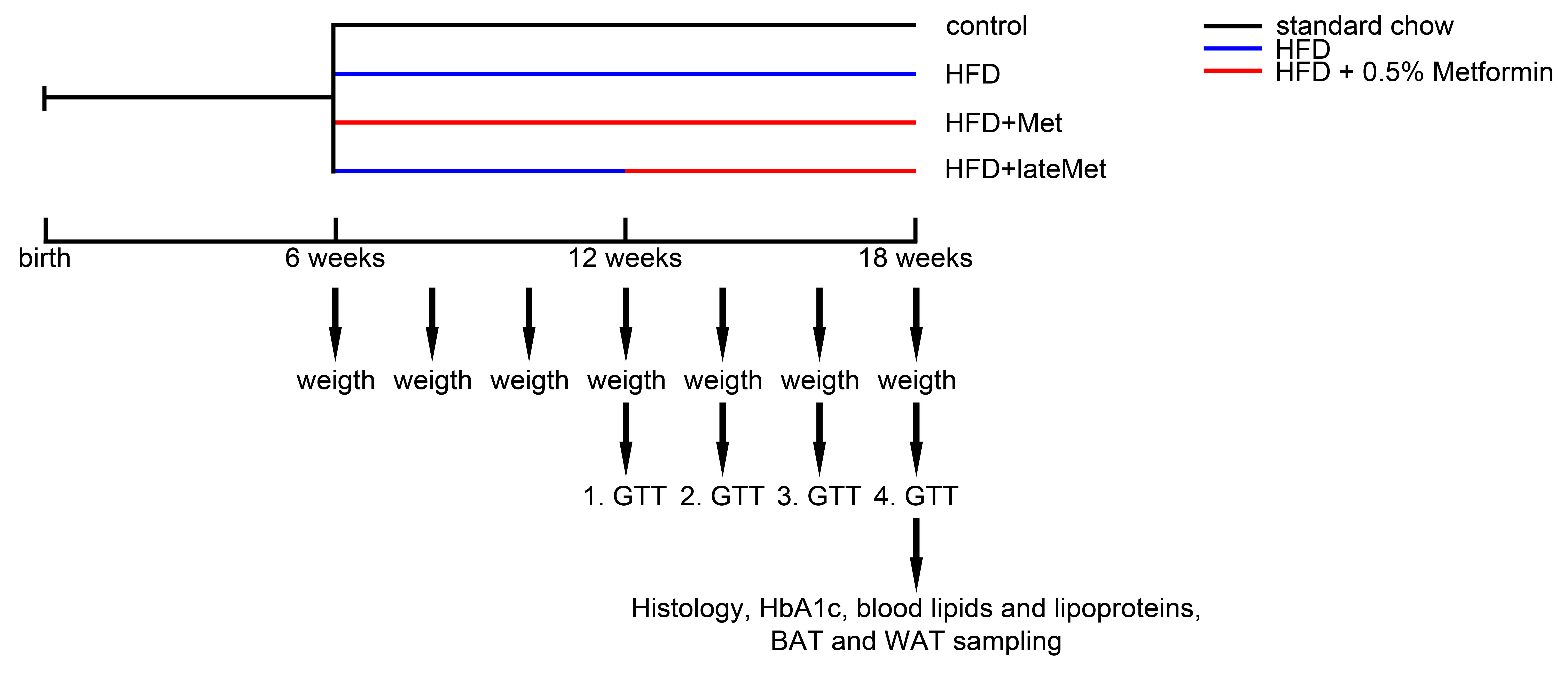


Supplementary Figure 1 – Treatment scheme.

The treatment scheme for control mice, which received standard chow, mice fed with high fat diet (HFD), mice fed with HFD and metformin starting at the same time (HFD+Met) and HFD mice treated with metformin after already developing glucose intolerance (HFD+lateMet) shows time points when weight measurement and intraperitoneal glucose tolerance tests (GTTs) were performed and when tissue and blood samples were taken.


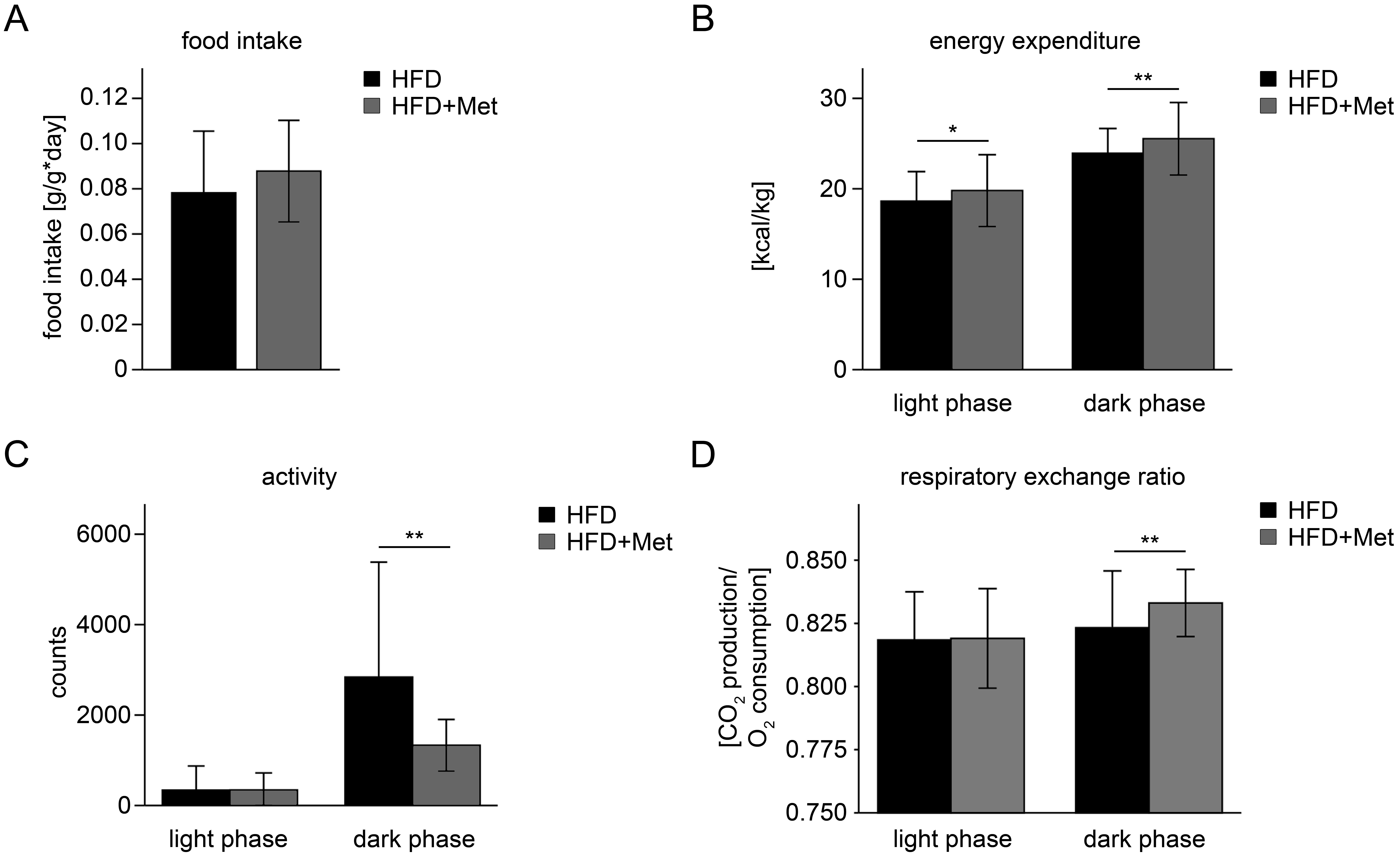


**Supplementary Figure 2** – Metformin increased energy expenditure and reduced physical activity**.**


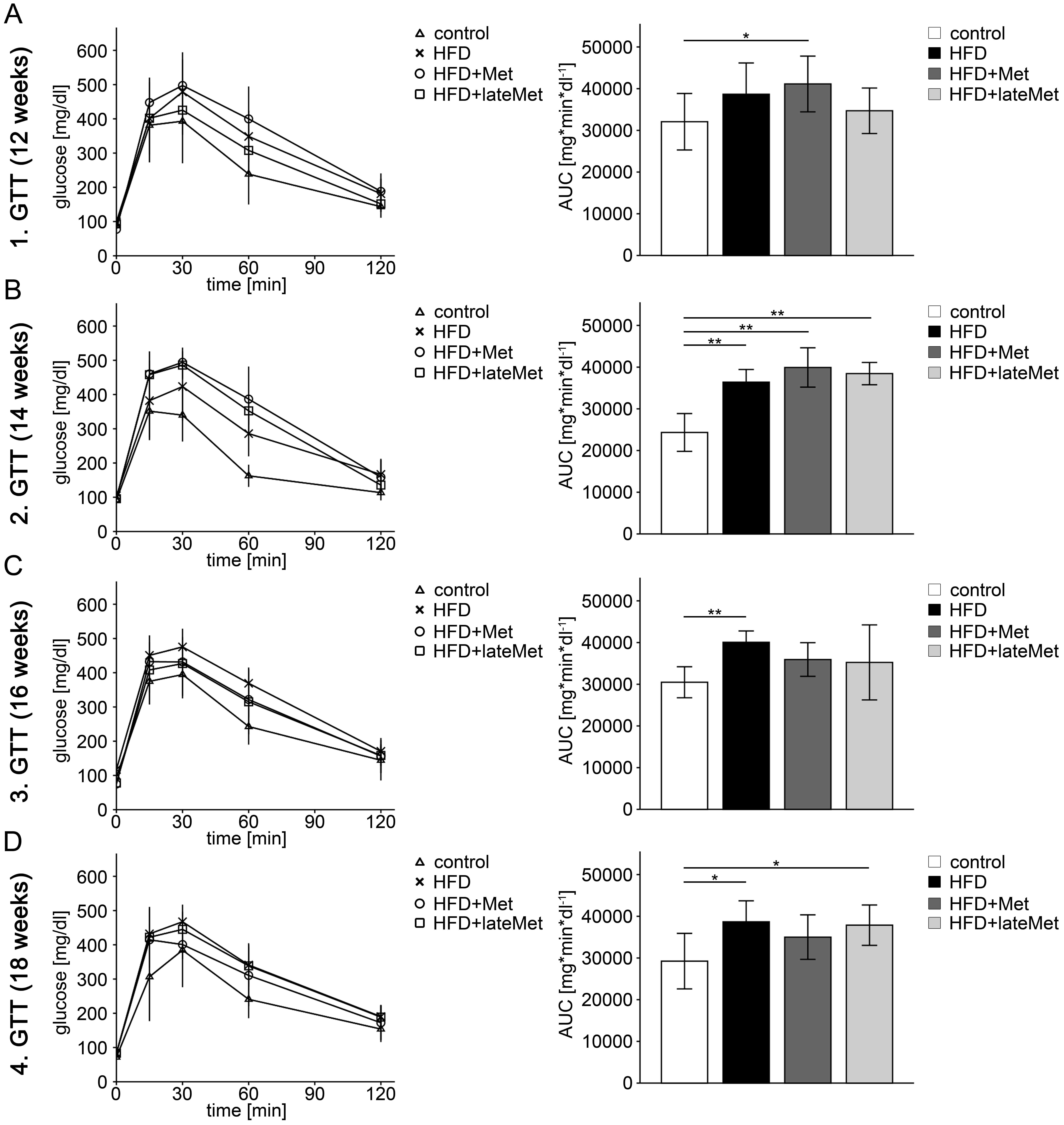


Supplementary Figure 3 – Intraperitoneal glucose tolerance tests (GTTs).

After (A) 12 weeks, (B) 14 weeks, (C) 16 weeks and (D) 18 weeks of treatment, GTTs of mice fed with high fat diet and treated with metformin starting at the same time (HFD+Met; n=11), HFD mice treated with metformin after already developing glucose intolerance (HFD+lateMet; n=8), HFD mice (HFD; n=7) and control mice (n=10), receiving standard chow, were shown on the left. On the right, calculations of AUC (area under the curve) were shown. There was impaired glucose tolerance in the HFD group starting at (B) 14 weeks and amelioration by metformin treatment at (C) 16 and (D) 18 weeks. Data are expressed as mean ± SD. n indicates the number of individual mice from which blood was collected and analyzed. Differences between groups were analyzed by 1-way ANOVA, followed by Bonferroni’s post-hoc test; *p<0.05 and **p<0.01.


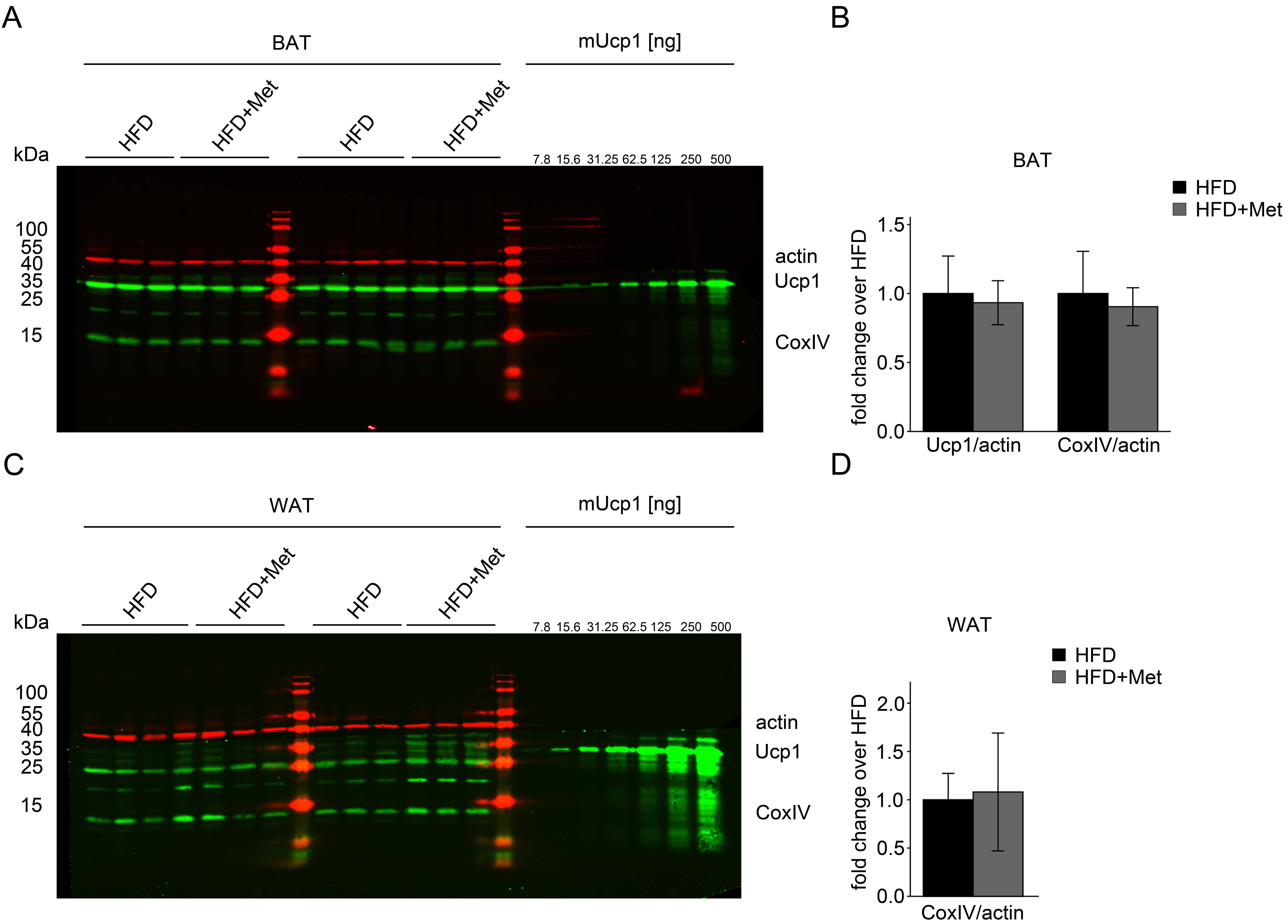


Supplementary Figure 4 – Expression of Ucp1 and subunit IV of cytochrome c oxidase.

(A+C) Representative immunoblots and (B+D) their densitometric analysis of uncoupling protein 1 (Ucp1) and subunit IV of cytochrome c oxidase in (A+B) brown adipose tissue (BAT) and (C+D) white adipose tissue (WAT) after 12 weeks of high fat diet (HFD; n=7) or HFD and metformin treatment starting at the same time treated with metformin starting at the same time (HFD+Met; n=6). Recombinant murine Ucp1 (mUcp1) was also loaded in order to facilitate identification of the correct band; Ucp1 is not present in detectable levels in WAT. Data are expressed as mean ± SD. n indicates the number of individual mice from which the fat tissue was collected and analyzed. Differences between groups were analyzed by Student’s t-test and found to be not significant.


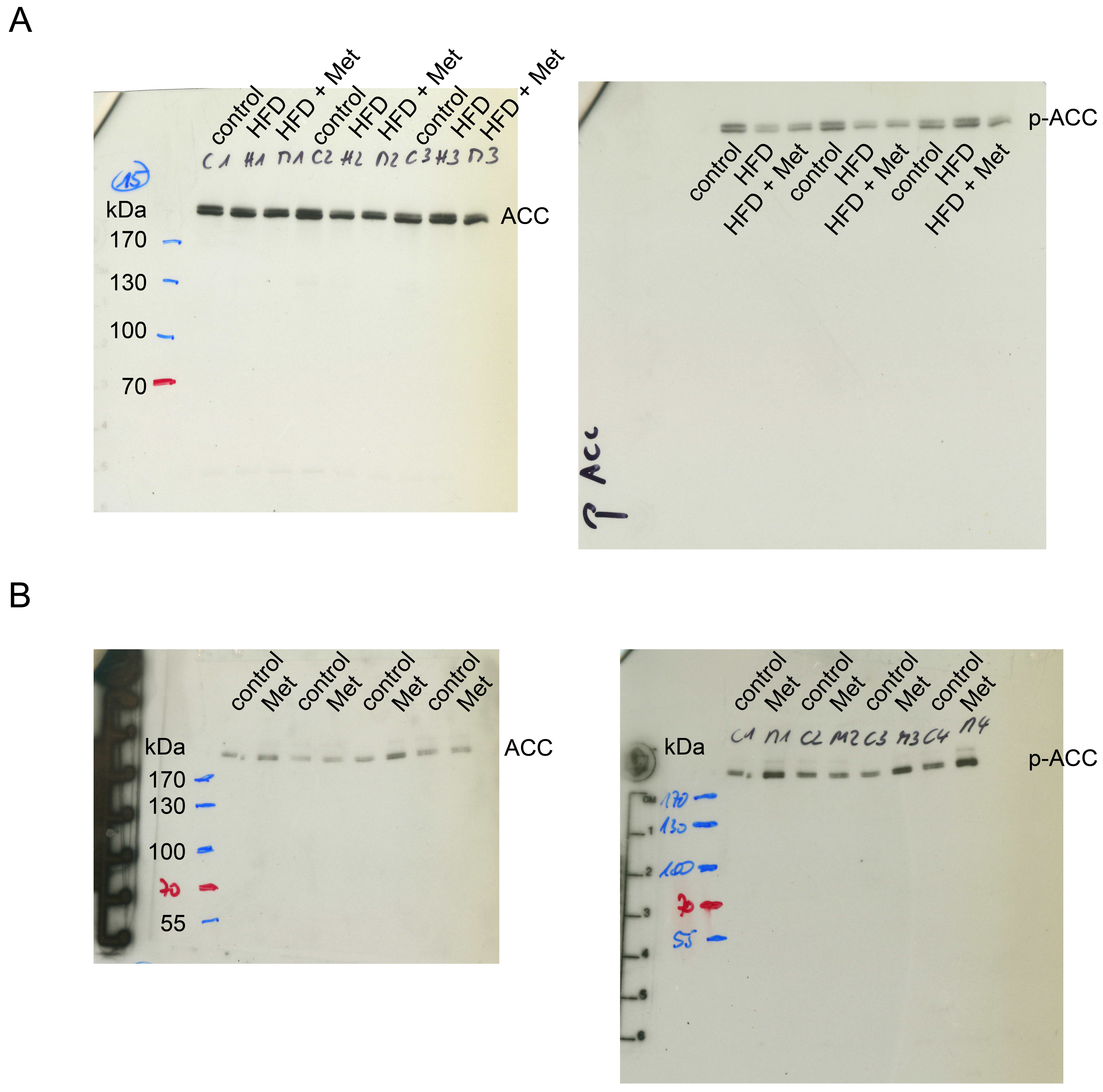


Supplementary Figure 5 – Original Western blots.

(A) Original unphosphorylated acetyl-CoA carboxylase (ACC) and at Ser 79 phosphorylated acetyl-CoA carboxylase (p-ACC) immunoblots of livers from mice fed with high fat diet and treated with 500 mg metformin kg^-1^ per day (HFD+Met), HFD mice (HFD) and control animals fed with standard chow after 12 weeks of treatment. (B) Original ACC and phosphorylated ACC (Ser79) immunoblots of livers of mice 3hours after giving 500 mg metformin kg^-1^ in one bolus by gavage and livers from non-treated control mice.


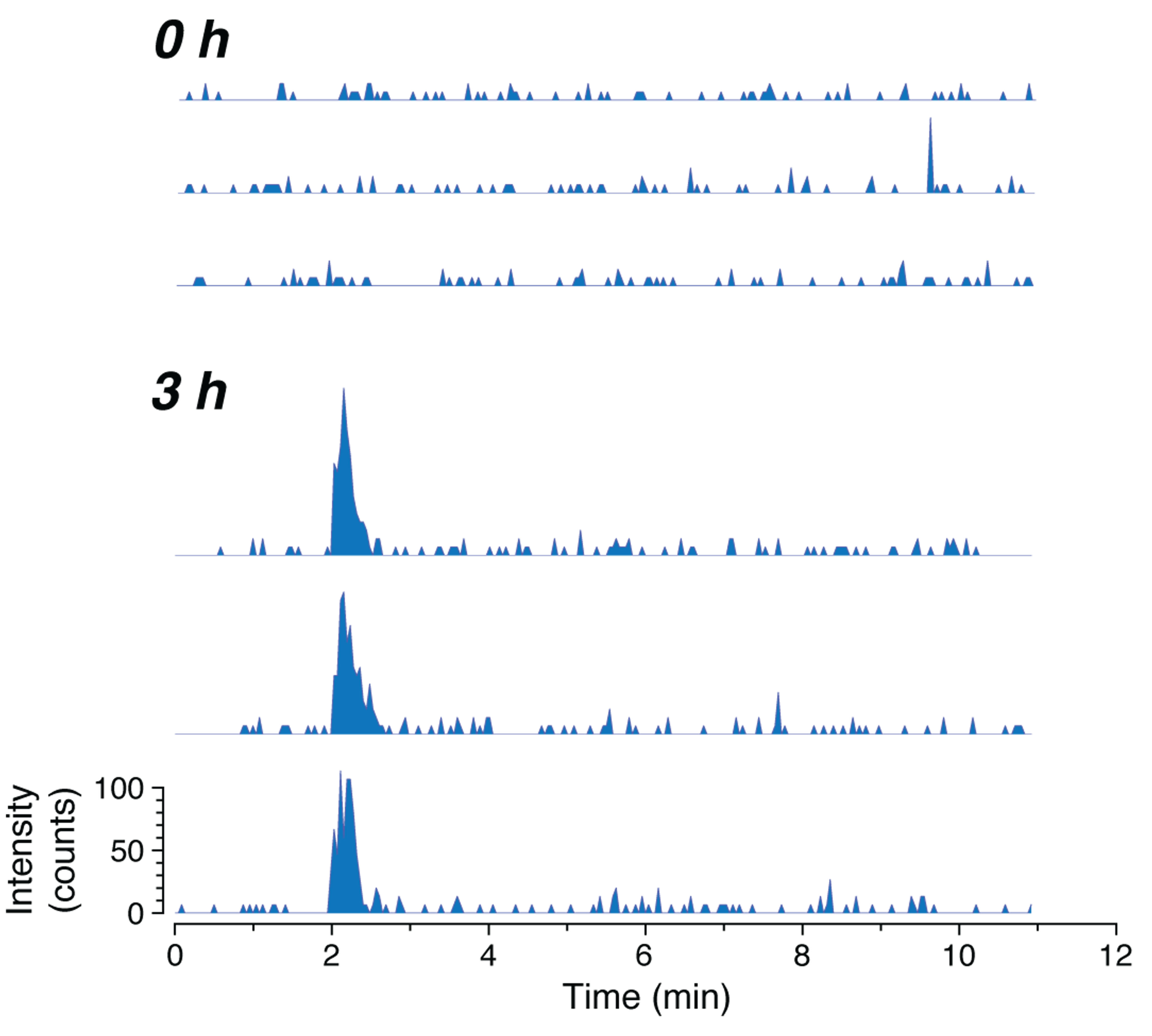


Supplementary Figure 6 – LC-MS/MS measurements

Robustness of LC-MS/MS measurement of glucose-1,6-13C in mouse plasma samples. The chromatograms show signal intensity (SRM, m/z 181 > 90; raw unsmoothed data) as a function of time for 3 mice on HFD without metformin, each at 0 h and 3 h feeding time (confer Fig. 4). The large peak at 2.2 min elution time represents glucose-1,6-13C.

## Supplementary Tables

|  | **Control** (n=8) | **HFD** (n=8) | **HFD+lateMet** (n=8) | **HFD+Met** (n=8) |
| --- | --- | --- | --- | --- |
| **Fasted Glucose** [mg/dl] | 72 ± 6 | 83 ± 12* | 83 ± 12* | 83 ± 12* |
| **HbA1c (electrophoretic)** [%] | 2,8 ± 0,2 | 3,1 ± 0,2* | 2,7 ± 0,2^#^ | 2,7 ± 0,1^##^ |
| **HbA1c (turbidimetric)**  [%] | 3,8 ± 0,3 | 4,2 ± 0,3 | 3,8 ± 0,4 | 3,6 ± 0,2^##^ |
| **HbA1c (calculated from turbidimetric analysis)** [mmol/mol] | 17.6 ± 2.5 | 22.4 ± 3.8* | 17.9 ± 4.5 | 15.6 ± 2.6^##^ |
| **Triglycerides** [mg/dl] | 14,3 ± 3,1 | 21,0 ± 6,9* | 30,2 ± 11,6* | 32,9 ± 12,2** |
| **Cholesterol** [mg/dl] | 17,2 ± 1,8 | 49,1 ± 12,7** | 41,6 ± 25,0* | 48,1 ± 15,8** |

**Supplementary Table 1** – **Metformin slowed down the development of a metabolic syndrome-like phenotype.**

After 12 weeks of treatment, sera was analyzed from the ventricle of mice fed with high fat diet and treated with metformin starting at the same time (HFD+Met; n=8), HFD mice treated with metformin after already developing glucose intolerance (HFD+lateMet; n=8), HFD mice (HFD; n=8) and control mice (n=8). In detail, fasted glucose, glycemic control, judged by glycated hemoglobin A1c (HbA1c) levels, triglycerides and cholersterol were measured. Data are expressed as mean ± SD. n indicates the number of individual mice from which blood was collected and analyzed. Differences between groups were analyzed by 1-way ANOVA, followed by Bonferroni’s post-hoc test; *p<0.05 and **p<0.01 HFD groups versus control; ^#^p<0.05 and ^##^p<0.01 HFD+Met or HFD+lateMet versus HFD. Regarding fasted glucose levels: 83 ± 12 mg/dl were indeed measured in HFD, HFD+lateMet and HFD+Met mice.

|  | **control** (n=6) | **HFD** (n=4) | **HFD+Met** (n=6) |
| --- | --- | --- | --- |
| **pH** | 7.24 ± 0.16 | 7.20 ± 0.07 | 7.25 ± 0.05 |
| **pCO_2_**[mmHG] | 57.9 ± 4.9 | 59.0 ± 10.8 | 57.1 ± 6.0 |
| **pO_2_**[mmHG] | 34.8 ± 6.5 | 36.5 ± 19.2 | 25.7 ± 2.0 |
| **Hb** [g/dl] | 15.4 ± 0.5 | 15.8 ± 0.4 | 14.6 ± 0.9 |
| **sO_2_** [%] | 39.6 ± 24.0 | 33.3 ± 23.56 | 23.0 ± 3.9 |
| **hematocrit** [%] | 47.1 ± 1.5 | 48.3 ± 1.2 | 44.9 ± 2.8 |
| **potassium** [mmol/l] | 4.9 ± 0.5 | 5.1 ± 0.4 | 5.2 ± 0.6 |
| **sodium** [mmol/l] | 141.0 ± 6.8 | 143.5 ± 5.3 | 146.5 ± 3.0 |
| **chloride** [mmol/l] | 113.8 ± 2.8 | 114.3 ± 1.7 | 113.7 ± 8.3 |
| **HCO_3_^-^** [mmol/l] | 24.3 ± 2.5 | 22.3 ± 1.7 | 23.9 ± 2.3 |
| **ABE** [mmol/l] | -3.8 ± 2.4 | -6.5 ± 2.4 | -3.5 ± 2.0 |
| **glucose** [mmol/l] | 7.7 ± 4.5 | 5.9 ± 2.6 | 8.9 ± 3.0 |
| **lactate** [mmol/l] | 3.6 ± 2.3 | 4.6 ± 2.2 | 4.0 ± 1.5 |

Supplementary Table 2 – No differences in blood parameters from the facial vein in response to 24 hours of metformin treatment.

After 24 hours of treatment, blood was analyzed from the facial vein of mice fed with high fat diet and treated with metformin (HFD+Met; n=6), HFD mice (HFD; n=4) and control mice (n=6). In detail, pH, partial pressure of carbon dioxide (pCO_2_), partial pressure of oxygen (pO_2_), hemoglobin (Hb), oxygen saturation (sO_2_), hematocrit, potassium, sodium, chloride, hydrogen carbonate (HCO_3_^-^), actual base excess (ABE), glucose and lactate were measured. Data are expressed as mean ± SD. n indicates the number of individual mice from which blood was collected and analyzed. Differences between groups were analyzed by 1-way ANOVA, followed by Bonferroni’s post-hoc test; p=n.s.

|  | **HFD** (n=4) | **HFD+Met** (n=6) |
| --- | --- | --- |
| **pH** | 7.24 ± 0.08 | 7.24 ± 0.10 |
| **pCO_2_**[mmHG] | 33.3 ± 7.0 | 24.8 ± 3.8 |
| **pO_2_**[mmHG] | 64.5 ± 5.5 | 75.2 ± 13.5 |
| **Hb** [g/dl] | 14.4 ± 1.2 | 13.7 ± 1.6 |
| **sO_2_** [%] | 76.2 ± 5.4 | 86.5 ± 5.4 |
| **hematocrit** [%] | 44.2 ± 3.7 | 41.9 ± 5.0 |
| **potassium** [mmol/l] | 5.6 ± 0.4 | 5.1 ± 0.6 |
| **sodium** [mmol/l] | 134.8 ± 11.1 | 139.3 ± 6.1 |
| **chloride** [mmol/l] | 111.5 ± 3.3 | 112.5 ± 4.5 |
| **HCO_3_^-^** [mmol/l] | 13.6 ± 2.5 | 10.6 ± 3.1 |
| **ABE** [mmol/l] | -12.8 ± 3.4 | -15.5 ± 5.2 |
| **glucose** [mmol/l] | 6.4 ± 2.9 | 3.8 ± 2.9 |
| **lactate** [mmol/l] | 7.2 ± 1.7 | 4.7 ± 3.6 |

**Supplementary Table 3 – No differences in blood parameters from the tail vein in response to 24 hours of metformin treatment.**

After 24 hours of treatment, blood was analyzed from the tail vein of mice fed with high fat diet and treated with metformin (HFD+Met; n=6) and HFD mice (HFD; n=4). In detail, pH, partial pressure of carbon dioxide (pCO_2_), partial pressure of oxygen (pO_2_), hemoglobin (Hb), oxygen saturation (sO_2_), hematocrit, potassium, sodium, chloride, hydrogen carbonate (HCO_3_^-^), actual base excess (ABE), glucose and lactate were measured. Data are expressed as mean ± SD. n indicates the number of individual mice from which blood was collected and analyzed. Differences between groups were analyzed by Student’s t-test; p=n.s.

| monocarboxylate-transporter 1  (Mct1; *Slc16a1*) | Forward 5’-tgttagtcggagccttcatttc-3’  Reverse 5’-cactggtcgttgcactgaata-3’ |
| --- | --- |
| monocarboxylate-transporter 4  (Mct4; *Slc16a3*) | Forward 5’-tcacgggtttctcctacgc-3’  Reverse 5’-gccaaagcggttcacacac-3’ |
| lactate dehydrogenase A  (*Ldha*) | Forward 5’‑tgtctccagcaaagactactgt‑3’  Reverse 5’‑gactgtacttgacaatgttggga‑3’ |
| lactate dehydrogenase B  (*Ldhb*) | Forward 5’‑cattgcgtccgttgcagatg‑3’  Reverse 5’‑ggaggaacaagctcccgtg‑3’ |
| glyceraldehyde-3-phosphate dehydrogenase (*Gapdh*) | Forward 5’‑atgtgtccgtcgtggatctga‑3’  Reverse 5’‑tgcctgcttcaccaccttct‑3’ |
| glucuronidase-β  (*Gusb*) | Forward 5’‑cgctgagagtaatcggaaacaa‑3’  Reverse 5’‑cgcaaaataaaggccgaagtgaagt‑3’ |

**Supplementary Table 4** – **Primers that were in use for RT-qPCR analyzes.**
